# Structural Study of a Peptide Epitope Bearing Multiple Post-Translational Modifications in Rheumatoid Arthritis

**DOI:** 10.3390/ijms26189026

**Published:** 2025-09-16

**Authors:** María José Gómara, Cristina García-Moreno, Oriol Bárcenas, Raúl Castellanos-Moreira, Juan Camilo Sarmiento, Ramon Crehuet, Yolanda Pérez, Raimon Sanmartí, Isabel Haro

**Affiliations:** 1 Unit of Synthesis and Biomedical Applications of Peptides, IQAC-CSIC, Jordi Girona 18-26, 08034 Barcelona, Spain; 2 Computational and Theoretical Chemistry Group, IQAC-CSIC, Jordi Girona 18-26, 08034 Barcelona, Spain; oriol.barcenas@iqac.csic.es (O.B.); ramon.crehuet@iqac.csic.es (R.C.); 3 Institut de Biotecnologia i de Biomedicina and Departament de Bioquímica i Biologia Molecular, Universitat Autònoma de Barcelona, 08193 Barcelona, Spain; 4 Department of Rheumatology, Hospital Clínic of Barcelona, 08036 Barcelona, Spainsarmiento@clinic.cat (J.C.S.); sanmarti@clinic.cat (R.S.); 5 NMR Facility, IQAC-CSIC, Jordi Girona 18-26, 08034 Barcelona, Spain; yolanda.perez@iqac.csic.es

**Keywords:** RA, α-fibrin (617–631) peptides, PTMs, AMPAs, CD, NMR, MD, conformation, structure

## Abstract

Given the limited knowledge of the effect of post-translational modifications (PTMs) on protein structure, in this study we investigated whether introducing one-to-three RA-related PTMs into the α-fibrin (617–631) peptide influences the conformation and structure of the peptide antigen that could be responsible for the autoantibody recognition. Ten peptides containing a different number of PTMs within their primary structure were synthesized and their recognition by sera from RA patients was analyzed. The conformation of the peptides was studied by circular dichroism (CD) and the structure of the most relevant antigenic peptides was determined by nuclear magnetic resonance (NMR) and enhanced-sampling molecular dynamics (MD). Although peptides containing citrulline (Cit) showed a higher degree of binding to AMPAs than peptides containing only homocitrulline and/or acetyl-lysine, the latter were able to bind to AMPAs in sera that showed a small response to peptides with Cit, with the response being different depending on the position of each PTM. CD and NMR analyses indicated a series of half-turn conformations in the Lys620-Arg630 region. MD simulations generated a set of conformations compatible with the NMR NOEs. The effect of the PTMs was observed in intra-molecular contacts, hydrogen bonds and van der Waals interactions, generating more collapsed conformations. Differences in autoantibody reactivity between peptides bearing different PTMs within their primary structures are noted. Peptides with PTMs adopt different conformations than unmodified peptides, probably due to the lower net charge of peptides with multiple PTMs, which may explain their recognition by autoantibodies.

## 1. Introduction

Rheumatoid arthritis (RA) is a systemic autoimmune disease that causes joint destruction and deformities, characterized by the presence of autoantibodies that have been implicated in the etiopathogenesis, diagnosis and prognosis of RA [1]. The autoimmune response that eventually occurs in genetically predisposed individuals to suffer RA [2] is triggered by post-translational modifications (PTMs) in certain proteins that drive the loss of tolerance to self-antigens. The best studied PTM in RA patients is citrullination, which consists of the conversion of positively charged arginine residues to the non-essential neutral amino acid citrulline in an enzymatic process mediated by the peptidylarginine deiminase family of enzymes (mainly the PAD2 and PAD4 isoenzymes) [3]. More recently other PTMs have been described in the context of RA, specifically homocitrullination and acetylation, in which modifications occur at lysine. These autoantibodies (anti-citrulline, anti-homocitrulline and anti-acetyl-lysine) are known as the anti-modified protein/peptide antibody (AMPA) family. The overlap between the three autoantibody families is characteristic of RA compared to other rheumatic diseases [4]. Structurally, PAD hydrolyses imino groups on arginine, thereby reducing the net charge of the proteins by losing one positive charge per modified arginine residue. This deimination process and the loss of charge can cause protein unfolding and disrupt intra- and inter-molecular interactions [5], resulting in a potentially autoantigenic structure. The deimination process can lead to the unfolding of α-helical protein structures [6,7] and, in other cases, can increase the percentage of β-sheet and aggregation into large insoluble amyloid fibrils [8] or cause protein polymerization [9]. Furthermore, the structural effects caused by homocitrullination and acetylation on self-proteins are similar to those of citrullination due to the loss of the positive charge on lysine residues. To date, studies of protein citrullination have shown that the degree and rate of modification of arginines to citrullines directly correlate with the structural order of the substrate [6,10], and are predicted to reside in solvent-accessible regions of the protein. Knowledge of the effect of citrullination/acetylation on protein structure is limited and is essentially based on circular dichroism (CD) measurements. The atomic-resolution structures of citrullinated proteins have not yet been determined experimentally. The AlphaFold 2 (AF2) algorithm [11] does not account for post-translational modifications that affect protein structure and function. In the context of rheumatoid arthritis, crystallographic studies have revealed how citrullination affects peptide binding to major histocompatibility complex (MHC) molecules and how this process may contribute to the development of autoimmune responses [12,13]. In recent years, several studies have presented an AlphaFold-based methodology for predicting the three-dimensional structures of peptide–MHC complexes [14,15], although it is not possible to introduce post-translationally modified peptide ligands.

Taking into account that the impact of PTMs on peptide conformation is a valuable but underexplored area, in the present work we attempt to examine how the antibody reactivity depends on this. With this aim, considering the ability and specificity of the α-fibrin (617–631) peptide sequence to recognize autoantibodies present in RA patients, which was widely described in previous works [16,17], here we analyzed the recognition of different versions of this peptide epitope according to the presence of different PTMs within its primary structure. As no three-dimensional experimental structures of the fibrin α-chain are available, we used AF2 structure prediction as a starting point. We found that the α-fibrin (617–631) domain belongs to a fibrin region with low complexity and is a solvent-exposed region with a low secondary structure. Therefore, one of the aims of this study is to investigate whether the introduction of one-to-three RA-related PTMs into the same peptide antigen influences the peptide conformation in solution which could be responsible for the observed autoantibody recognition. The most relevant antigenic peptides were studied by CD and nuclear magnetic resonance (NMR) spectroscopy in the presence of trifluoroethanol (TFE) and NOE-derived molecular dynamic distance restraints in order to obtain a set of conformations compatible with the NMR data. As reviewed in the literature [18], TFE excludes water from peptide surfaces and decreases the dielectric constant of the medium, creating a more hydrophobic environment similar to that formed when a peptide–protein complex is created.

## 2. Results and Discussion

**Figure 1:**
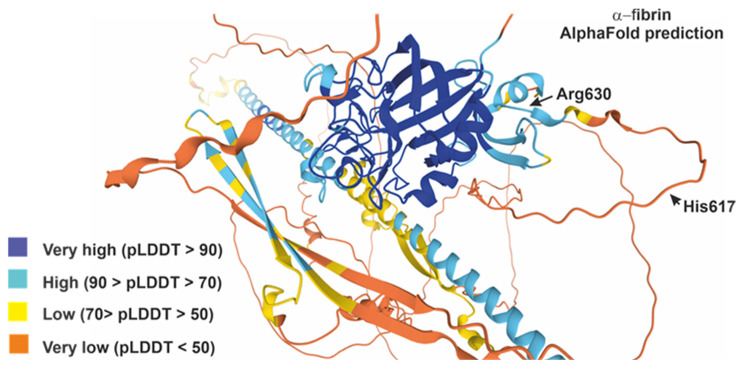
Predicted structure of full-length fibrin α chain by AlphaFold (AF-P02671-F1), colored by pLDDT value (from lowest to highest: orange, yellow, light blue and dark blue).

According to AF2 prediction for the α-fibrin protein, the (617–631) domain is located in a solvent-exposed region with a low to very low secondary structure (70 > pLDDT > 50). This region consists of a protein loop with a mostly random coil structure, except for some β-turn-like structures between Gly622 and Arg630 (see Figure 1), and comprises two lysines and three arginines. Therefore, we considered the selected α-fibrin (617–631) peptide as a suitable candidate with which to examine the influence of peptide backbone conformation on antibody reactivity based on the presence of different PTMs within its primary structure.

### 2.1. Peptide Synthesis

The 15-mer fibrin peptide, HSTKRGHAKSRPVRG [α-fibrin(617–631)], was used as a template to generate ten peptides with varying numbers of PTMs at various positions and subsequently to study the effect of these PTMs on AMPA antibody binding from RA patient serum. Considering that the biotin–avidin interaction is one of the strongest and most stable non-covalent interactions known, as well as the results of the previously published work by Babos et al. [19] where the authors studied the role of N- or C-terminal biotinylation in autoantibody recognition, the set of synthetic peptides were N-terminally biotinylated. As established by these authors, the position of biotin should not affect the accessibility of the epitope core or the detection of the antibody binding, since in our conjugates the relevant Cit^630^ is located near the C-terminus. Furthermore, the distance between the epitope binding site and the biotin was reinforced by the incorporation of two PEGs. All biotinylated peptides were successfully synthesized by Solid-Phase Peptide Synthesis (SPPS) and subsequently characterized by analytical High-Performance Liquid Chromatography (HPLC) and Electrospray Mass Spectrometry (ESI-MS) (Table 1 and Figure S1). After HPLC purification the purity was in all cases greater than 95%. The overall yield of synthesis and purification of these peptides ranged between 30 and 50%.

### 2.2. Recognition of the Different Epitopes by RA Patients’ Sera

**Figure 2:**
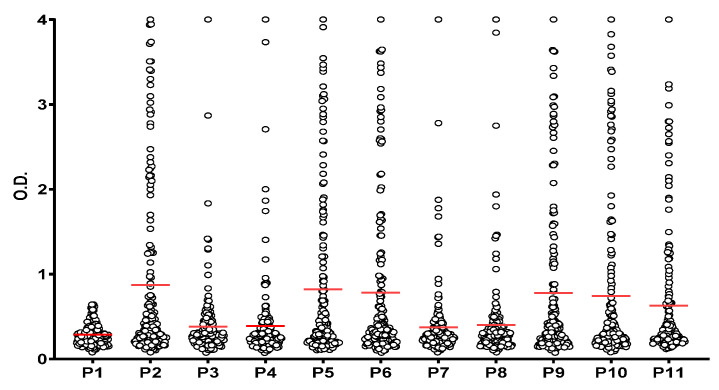
Reactivity of RA patients’ sera (n = 172) to α-fibrin(617–631) peptides. Significant antibody reactivity (*p* ˂ 0.05) was found to all PTM-derived peptides (P2–P11) compared to the control peptide (P1). Red lines indicate the mean of all O.D. values for each peptide which are represented in circles.

First, we compared the reactivity of the RA patients’ sera against the native fibrin peptides to ensure that the reactivity to each of the other ten PTM-containing peptides was specific to the presence of each combination of citrulline, homocitrulline and acetyl-lysine. Figure 2 shows that a significant antibody reactivity (*p* ˂ 0.05) was found for all PTM-modified peptides (P2 to P11) compared to the control peptide (P1). The peptides were then divided into two groups based on the number of PTMs presented: one group contained three versions of α-fibrin(617–631) with a single PTM, namely Cit (P2), hCit (P3) or KAc (P4), and the other group contained seven combinations of two or three PTMs (P5 to P11). Differences in autoantibody reactivity between control peptide (P1) and PTM-derived peptides were calculated by unpaired *t*-test. Analyses were performed in Graph Pad Prim 8.0.2.

### 2.3. Reactivity to Peptides Containing a Single PTM

**Figure 3:**
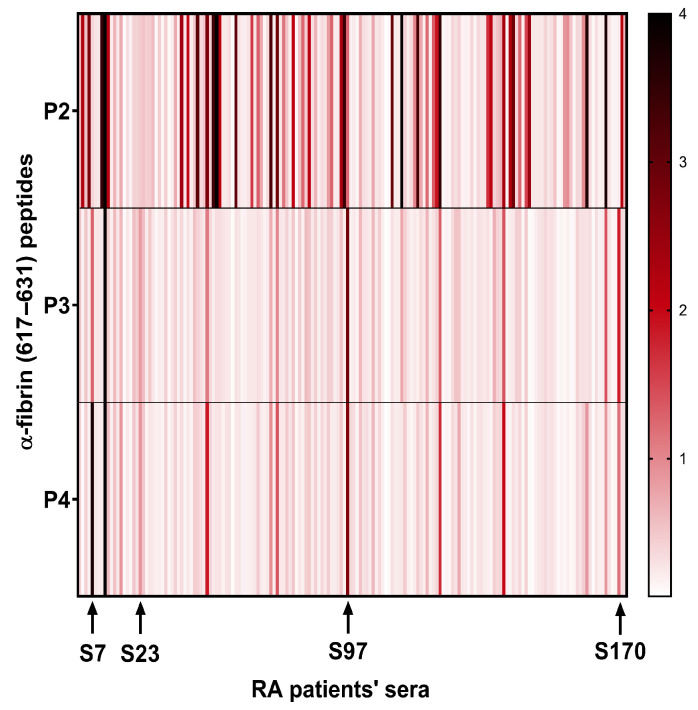
Heat map of the optical density (OD) reactivity of RA patient sera to peptides containing a single PTM. The arrows highlight sera with little or no reactivity to the citrulline peptide(P2) but reactive to the homocitrulline (P3) and/or acetyl-lysine (P4) peptides.

The citrullinated α-fibrin(617–631) peptide (P2) was recognized by a greater number of RA patients’ sera and showed significantly higher binding to AMPAs in terms of mean intensity compared to its homocitrullinated and acetyl-lysine counterparts (P3 and P4). However, interestingly, some sera from this collection (S7, S23, S97 and S170) showed reactivity to the homocitrullinated and/or acetyl-lysine versions of the peptide but not to the citrullinated peptide, suggesting that each post-translational modification might generate a unique and independent antibody response (Figure 3).

### 2.4. Recognition of Peptides Containing Multiple PTMs

To further evaluate the effect that each PTM has on epitope recognition by AMPAs present in RA patients’ sera, peptides containing two or three modifications simultaneously were used. Analysis of the reactivity detected to these peptides (P5 to P11) revealed that the citrulline-containing peptides (P5, P6, P9, P10 and P11), exhibited a higher degree of AMPA binding than the two other peptides lacking this post-translational modification in their structure (P7 and P8). However, essentially no significant difference was detected among all the epitopes containing at least one citrulline within their structure (Biotinyl-PEG_2_-HSTXXGHAXSRPVCitG) as mean OD values of peptides containing two citrullines or the epitope containing only one citrulline were similar (P6, P9 and P10 vs. P11 *p* ˃ 0.05; P5 vs. P11 *p* = 0.037). In agreement with these results and as previously reported, the presence of Cit in position 630 surrounded by small neutrally charged amino acids (specifically the Cit-Gly motif) appears to be essential for antibody recognition [17,20].

**Figure 4:**
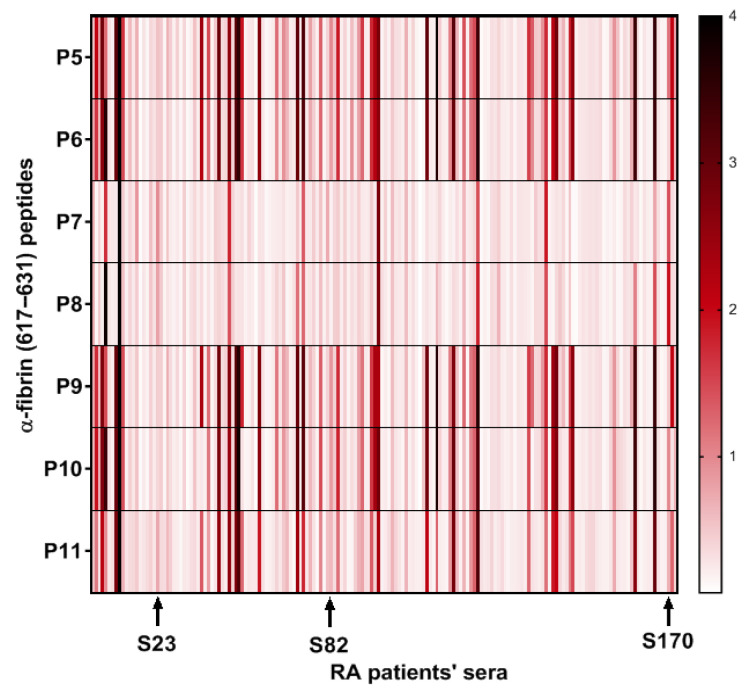
Heat map of the optical density (OD) reactivity of RA patient sera to peptides with multiple PTMs. The arrows highlight sera with low reactivity to citrulline-containing peptides (P5, P6, P9, P10 and P11) but with higher reactivity to non-citrulline peptides (P7 and P8) in terms of OD.

On the other hand, P7 and P8, epitopes containing only homocitrulline and acetyl-lysine as PTMs, despite showing a lower overall reactivity, were able to bind to AMPAs from sera that showed a small response to Cit-containing peptides, which is in agreement with the previous finding observed for the peptides P2, P3 and P4 that presented a single PTM in their sequences. Interestingly, the response to P7 and P8 detected in these sera (S23, S82, S170) was not identical, suggesting that both the presence and the position of each PTM are critical for antibody recognition (Figure 4).

### 2.5. Conformational and Structural Studies

#### 2.5.1. Circular Dichroism Conformational Studies

**Figure 5:**
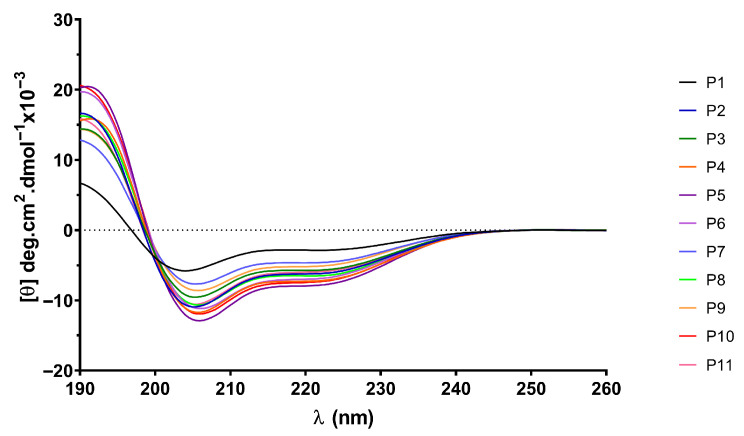
CD spectra of P1–P11 peptides in TFE.

In order to monitor the secondary structure of the peptides and to analyze whether the incorporation of different PTMs into the parent sequence (P1) could be related to the observed antigenicity, CD spectra were recorded. Since the ELISA assays were performed in aqueous medium, the CD spectra of P1-P11 were initially compared using aqueous peptide solutions. In all cases, an intense negative band around 200 nm and a weak negative shoulder at λ~230 nm were observed, indicating a random coil conformation for the biotinylated peptide sequences independently of the type and content of PTMs in their primary structure, as well as the net charge of the peptides at neutral pH (Figure S2). However, when the analysis was carried out using halogenated alcohol (TFE) as solvent, characteristic bands located at 205 and 222 nm appeared, showing that the spectra were more similar to those typical of an α-helix structure (Figure 5). The estimate of the α-helix content based on the molar ellipticity at 222 nm [21,22] was between 9 and 24% (Table S1), with the lowest contribution to the helical structure being observed for the peptide with no PTMs in its sequence (P1). No marked differences were found in the spectral pattern between peptides containing the different combinations of PTMs (P2–P11 peptides) and only minor differences in the α-helix content were observed. However, although the incorporation of PTMs into the native fibrin peptide did not significantly alter the secondary structure observed in TFE, it clearly exhibited a less ordered structure in the absence of PTMs compared to the presence of multiple PTMs. This result may suggest that the conformation of the peptide influences its recognition by autoantibodies.

#### 2.5.2. Nuclear Magnetic Resonance Structural Studies

Short linear peptides in solution exist as ensembles of structures that transiently interconvert and rapidly exchange. Therefore, both CD and NMR data represent a population-weighted average of all conformers. As can be seen in Figure 5, the CD results suggest that secondary structure appears to be present at different levels ranging from 9 to 24% (Table S1), depending on the peptide, in the presence of 100% TFE. In the simplest case, these peptides undergo conformational averaging between a single structure and a random coil state in the presence of TFE. We therefore carried out a conformational study of some of these peptides by solution NMR spectroscopy. We selected P1, P2 and P10 considering that P1 was the control peptide (without PTMs in its sequence); P2 was the citrulline-containing α-fibrin version (617–631) (with 2 Cit in its sequence) that was recognized by a higher number of RA patients’ sera; and P10 was the α-fibrin version (617–631) containing the three RA-relevant PTMs (Cit, hCit and AcK) that showed higher AMPA binding. The ^1^H NMR spectra of peptides P1, P2 and P10 in water or TFE/water are shown in Figures S3–S6. For P2, the addition of 30% TFE results in the NH chemical shifts of most residues moving upfield, with the exception of Ser2 (the most downfield amide signal at 8.85 ppm), the side-chain aromatic proton resonances of His1 and His7 and the side-chain NH resonances of Lys4, Lys9 and Arg11, which move downfield. The downfield shift in resonances in residues with OH/NH groups in the side chain can be attributed to the formation of hydrogen bonds between these groups and TFE. Furthermore, TFE can bind to the oxygenated carbonyl group of the main chain, which favors the formation of intrapeptide hydrogen bonds of the amide group, since the amide hydrogen is less exposed to the solvent. Further addition of TFE-d_2_ (50:50 *v*/*v*) shifts these resonances further up/down, respectively. For P10, the shift in these side-chain resonances upon addition of TFE is minor (compare the shifts in the His1/His7 CHε resonances in Figures S3 and S4). Unlike peptides P2 and P10, further addition of TFE (from 30% to 50%) does not significantly affect the chemical shift in peptide P1 (see Figure S5). Figure S6 compares the amide region of the ^1^H NMR spectra of peptides P1 (red), P2 (green) and P10 (blue) in a 70:30 (*v*/*v*) mixture of H_2_O and TFE-d_2_. Previous studies have shown that gradually adding TFE to short peptides results in a progressive decrease in contacts between the peptide and water molecules. This confines the TFE mainly to the first solvation shell around the peptide, resulting in an almost complete absence of peptide–water contacts at a TFE:H_2_O ratio of 70:30 (*v*/*v*) [18]. The problem with higher amounts of TFE (>60% *v*/*v*) is that it preferentially stabilizes proteins and peptides in a helical conformation, regardless of their native structural propensities. As the proton NMR spectra of the three peptides did not show any overall changes when the TFE concentration was increased from 30% to 50%, we selected the minimum amount of TFE (70:30 (*v*/*v*) TFE:H_2_O) required to create a more hydrophobic environment similar to that formed when a peptide–protein complex is created.

**Figure 6:**
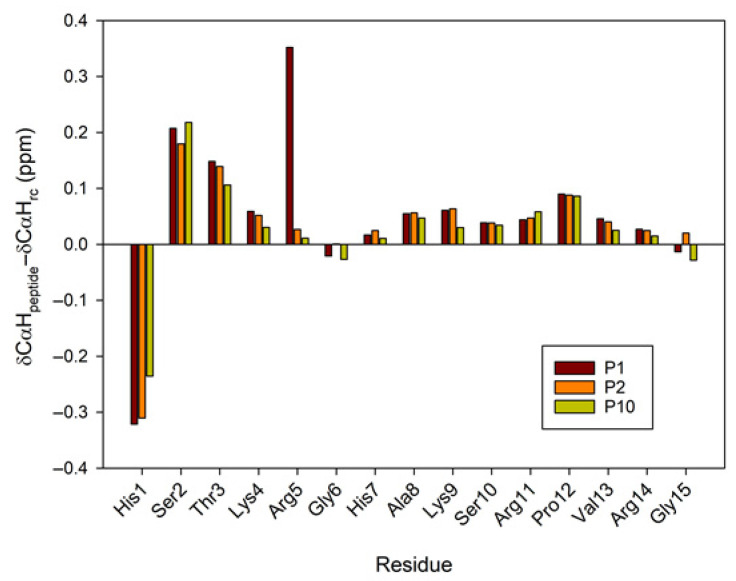
Conformational shift maps the of Cα protons for the three selected peptides in H_2_O/TFE-d_2_ (70:30 *v*/*v*). The difference between the observed proton chemical shifts and the random coil chemical shifts defined in [24,25,26] is plotted as a function of residue number for each peptide. A positive shift indicates a shift to a lower field than the random coil shift.

**Figure 7:**
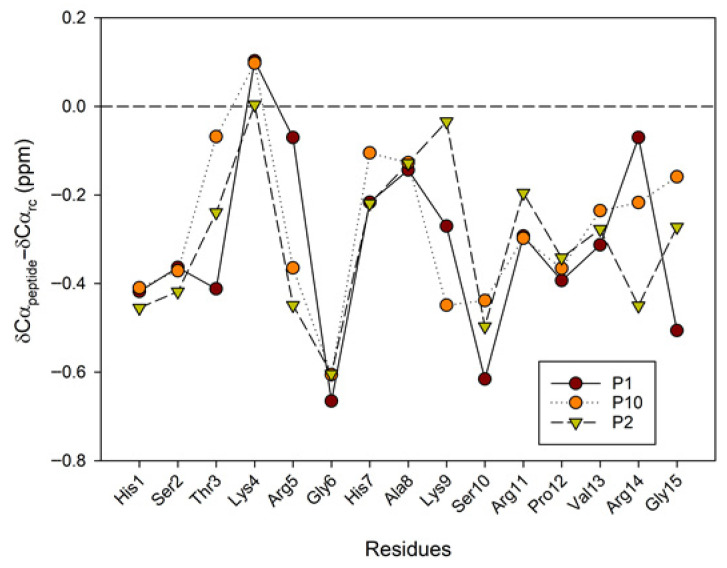
Conformational shift maps of the ^13^Cα carbon for the three selected peptides in H_2_O/TFE-d_2_ (70:30 *v*/*v*). The difference between the observed Cα chemical shifts and the random coil chemical shifts defined in refs [24,25,26] is shown as a function of the number of residues in each peptide.

**Figure 8:**
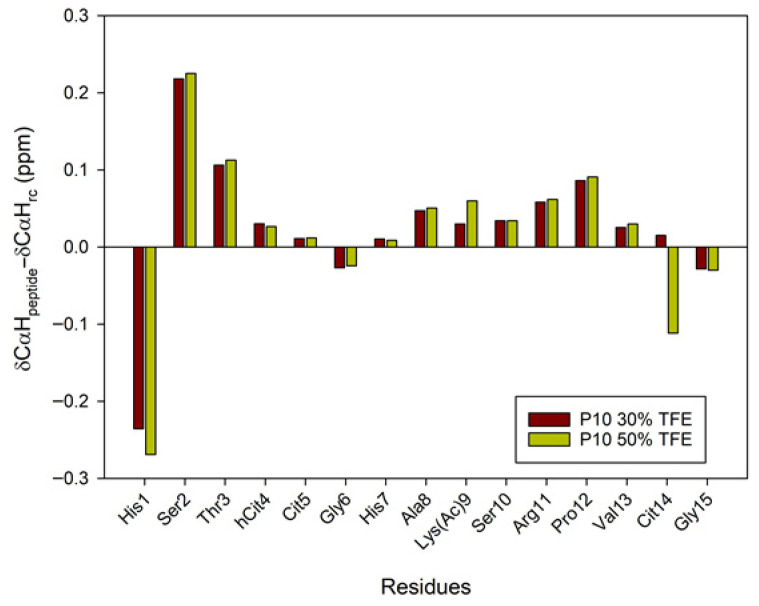
Conformational shift maps of the Cα protons of peptide P10 in H_2_O/TFE-d_2_ (70:30 and 50:50 *v*/*v*) are shown. The difference between the observed proton chemical shifts and the random coil chemical shifts defined in [24,25,26] is plotted as a function of the number of residues in each peptide.

A large body of research has demonstrated the significant contribution of regular secondary structure to chemical shift values in peptides and proteins [23]. The conformational shifts in Cα protons or ^13^Cα carbons can be obtained by subtracting the random coil chemical shift values [24,25] from the experimental values. Therefore, to advance the investigation of the conformational behavior of selected peptides in 30%/50% TFE (NMR), we evaluated the conformational shifts of Δδ_CαH_ and Δδ_Cα_. The established random coil values were corrected due to the presence of modified residues, such as citrullinated or acetylated residues [26]. Figure 6 and Figure 7 show the conformational shifts in Hα and Cα (Δδ = δ_observed_ − δ_random coil_) in 30% TFE for the three peptides. Figure 8 shows the conformational changes in Cα at 30% and 50% TFE for peptide P10. All three peptides exhibit similar chemical shifts along their sequences in the presence of TFE. However, the Hα at position 5 shifts substantially upon the Arg to Cit change. Published studies show that Cα protons shift downwards in β-sheet regions, whereas helical regions shift upwards. Cα carbons and carbonyl shifts also show a strong correlation with secondary structure, shifting downwards in helical conformations and upwards in β-strand or extended configurations [23,27,28,29,30]. However, analysis of the Δδ_CαH_ and Δδ_Cα_ profiles of 30% TFE of the three peptides does not align with the changes expected for an α-helix (Δδ_CαH_-negative and Δδ_Cα_-positive; Figure 6, Figure 7 and Figure 8), suggesting the presence of a β-turn or hairpin in certain amino acid sequences [31,32,33,34].

CD spectra show that peptides dissolved in 100% TFE exhibit a strong positive absorbance below 195 nm and a double-minimum negative ellipticity between 205 and 222 nm (Figure 5). This could indicate a quantifiable population of an α-helical structure. However, we must consider that type I turns also present α-helix-like CD spectra, with negative bands around 220 and 210 nm, as well as a positive band around 190 nm [31,35]. Therefore, the CD spectra shown in Figure 5, obtained at 100% TFE, could be due to an α-helix or a reverse β-I- or β-III-type turn [35,36]. Although most examples in the literature demonstrate the formation of α-helices, other structures such as turns, β-hairpins, β-sheets, hydrophobic clusters and associated surfaces are also stabilized in the presence of TFE [37,38]. According to the AF2 prediction for the protein α-fibrin, the region (617–631) consists of a protein loop with a mainly random coil structure, with some β-turn-like structures present between Gly622 and Arg630 (see Figure 1). In addition to the CD results and the positive conformational changes in the Hα protons and negative values of the Cα carbons, which could indicate turn conformations spanning these residues [31,32,33], we observe a series of half-turns in the Lys620–Arg630 region (Lys4 and Arg14 in our peptides) of the Alphafold structure of the parent α-fibrin protein (Figure 1). To complete the conformational analysis, we acquired 2D ^1^H-^1^H NOESY spectra to investigate the structural properties of peptide P10 in water and peptides P1, P2 and P10 in a water/TFE-d_2_ (70:30 *v*/*v*) solution at pH ~3 (Figures S7–S9). The NOESY experiment on peptide P10 in water yielded few overlapping correlations, so proton chemical shift assignment was not possible. In 30% TFE, most peptide NOE correlations were intra-residue and sequential between backbone protons. However, several inter-residue correlations were also observed. For an α-helix, strong mid-range NOEs are known to be observed in succession (dNN(i, i + 2), dαN(i, i + 3) and dαβ(i, i + 3)) and weak long-range NOEs (dαN(i, i + 4)) [39,40]. For example, some distinctive correlations were identified during the search for non-overlapping mid-range NOEs for peptide P2 in 30% TFE: one dαβ(i, i + 3) Ser2-Cit5; one dαβ(i, i + 3) Arg11-Cit14; and two dαN(i, i + 3) Ser10-Val13 and Arg11-Cit14. The remaining observed NOE correlations were short-range (intra- and inter-residue NN, αN and βN(i, i + 1)). Therefore, the solvent-induced non-native secondary structure appears to persist in pure TFE, as demonstrated by CD experiments. However, distance data in semi-aqueous conformations (30–50% TFE) are more difficult to interpret. This is partly because several correlations must be discarded due to overlapping resonances and partly because of the presence of a conformational ensemble in an aqueous solution. Therefore, to clarify the discrepancies/ambiguities between the experimental CD and NMR data, we decided to use the strengths of the ^1^H-^1^H NOE correlations to obtain upper-bound distance restraints. The final set of restraints obtained (intra-residual, sequential, mid-range and long-range NOEs) will be used to calculate the structural ensemble P1, P2 and P10 in 70:30 H_2_O:TFE by molecular dynamics. Thus, a non-native solvent-induced secondary structure appears to persist in pure TFE (CD experiments), but the data are more ambiguous in a semi-aqueous conformation (NMR, 30–50% TFE) and are compatible with some types of turn structure [34]. Therefore, to clarify the discrepancies and ambiguities between the experimental CD and NMR data, we use ^1^H-^1^H NOE correlation strengths to obtain upper-bound distance restraints. The final set of obtained restraints (intra-residual, sequential, mid-range and long-range NOEs; Tables S2–S4) will be used to calculate the ensemble of P1, P2 and P10 structures in a 70:30 H_2_O:TFE mixture by molecular dynamics (MD) simulations.

#### 2.5.3. Molecular Dynamics Simulations

**Figure 9:**
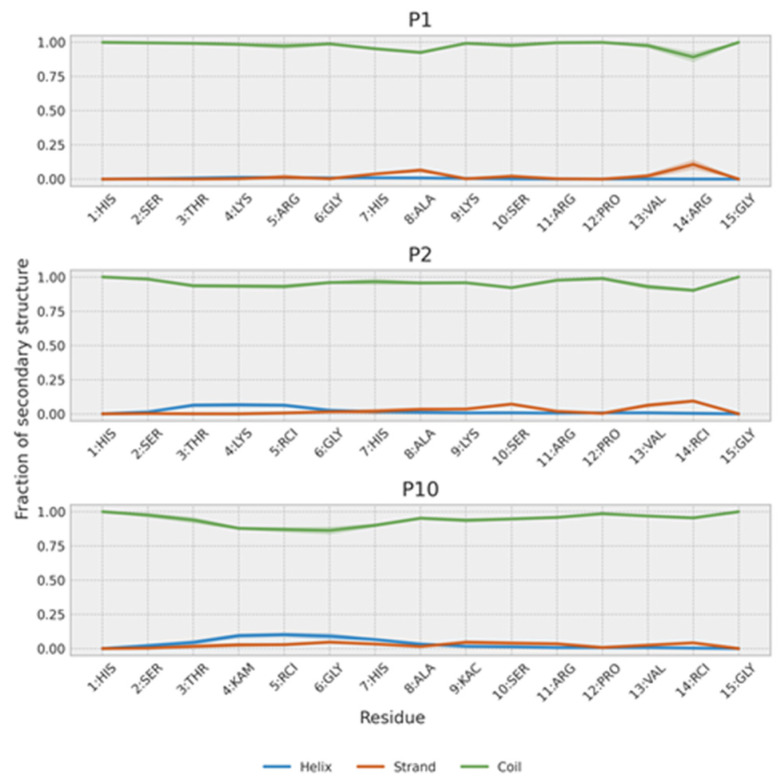
Secondary structure population of P1, P2 and P10. The shaded region represents the estimated error of the mean.

**Figure 10:**
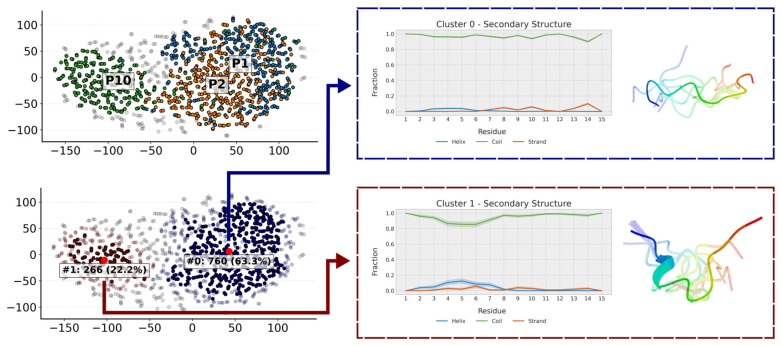
Clustering of the trajectories of P1, P2 and P10. The populations of each cluster correspond to the aggregated conformations of the three trajectories, such that each individual trajectory has a population of 33.3%. Clusters of this size indicate that the entire trajectory is grouped within that cluster, highlighting the conformation of the cluster centroid. When coloring the clusters by peptide (P1 in blue, P2 in orange and P10 in green) one can see that P10 populates a cluster of more compact conformations almost on its own. The presentative conformation of each of the clusters has also been represented.

We performed molecular dynamics simulations of P1, P2 and P10 in the same solvent conditions as the experimental NMR (70:30 (*v*/*v*) TFE:H_2_O) to generate a set of conformations compatible with the NMR NOEs. The results of these simulations are visualized in Figure 9 and Figure 10 and show that these three peptides adopt different conformations. Back-calculation of chemical shifts for these trajectories shows a reasonable agreement with the experiment and, most importantly, that the all three peptides are described with equal accuracy, without biasing the secondary structure for any of them (Figure S10).

In agreement with NMR results, the simulations show that all three peptides weakly populate secondary structure conformations (Figure 9). Further analysis of their conformations can be obtained by clustering the trajectory conformations. As expected for an unstructured peptide, clusters are diffuse with considerable overlap (Figure 10). However, two clusters can be defined. One cluster is populated mainly by P1 and P2, and, to a lesser extent, by P10. This cluster is composed of extended random-coil conformations. A second cluster is almost only populated by P10 and corresponds to conformations that have a small amount of helical structure and are more compact in the N-terminal region. That explains the gradual decrease in the radius of gyration from P1 (1.04 ± 0.22 nm) to P2 (0.93 ± 0.21 nm) and P10 (0.87 ± 0.21 nm). These more collapsed conformations result in some turns that may favor transient β-strands, as is slightly seen mainly in P2 (Figure 9). The effect of the PTMs is also observed at intra-molecular contacts (Figure S11). Citrullination of Arg5 increases its contacts, mainly with His1 and Ser2 in P2 and, to a lesser extent, in P10. Acetylation of Lys9 in P10 favors contacts with His7. The i, i + 3 and i, i + 4 contacts are typical of α-helices and are more prevalent in P10. P2 shows more contacts than P1 because it is more compact, especially in the C-term region. This region is similar to the P10 region, as expected because they share the same sequence.

## 3. Materials and Methods

### 3.1. Synthesis, Purification and Characterization of Peptides

Ten novel peptide sequences based on the primary structure of the α-fibrin (617–631) region containing a single post-translational modification (PTM) or combinations of two or three different PTMs (citrulline -Cit-, homocitrulline -hCit- and acetyl-lysine -KAc-) and a control peptide with no PTMs were designed and synthesized by SPPS. Each of the peptides was manually synthesized as a C-terminal carboxamide on a TGR-R resin in a polypropylene syringe fitted with a porous polyethylene disk following a 9-fluorenyl-methoxycarbonyl (Fmoc) strategy. Amino acid side-chain protection was affected by the following: triphenylmethyl (Trt) for histidine; tert-butyl (tBu) for serine and threonine; 2,2,5,7,8-pentamethyl-chroman-6-sulfonyl (Pmc) for arginine and tert-butoxycarbonyl (Boc) for lysine. Coupling reactions were performed using three-fold molar excesses of Fmoc-L-amino acids activated by treatment with 2-(1H-7-azabenzotriazole-1-yl)-1,1,3,3-tetramethyluronium hexafluorophosphate methanaminium (HATU) and diisopropylethylamine (DIPEA) throughout the synthesis. Removal of the Fmoc group from each amino acid was accomplished by means of treating the resin with 20% (*v*/*v*) piperidine in dimethylformamide (DMF) twice for 10 min. All coupling and deprotection steps were evaluated using the Kaiser colorimetric assay, based on the reaction of ninhydrin with primary amines or the chloranil test that allows the detection of secondary amino groups. Once the elongation of each peptide sequence was completed, a part of the peptidyl-resin was biotinylated at the N-terminus. The biotinylation step was completed by adding N-biotinyl-NH-(PEG)_2_-COOH (4 equivalents) dissolved in a minimal volume of N-Methyl-2-pyrrolidone in the presence of a phosphonium salt (benzotriazole-1-yloxytris(pirrolidino) phosphonium hexafluorophosphate (PyBOP, 4 equivalents), as well as 1-hydroxybenzotriazole (HOBt, 4 equivalents) and a DIPEA (8 equivalents). The reaction was left overnight and its completion was checked by the ninhydrin colorimetric reaction. Peptides were cleaved from the resin after elongation was completed by means of treatment with 95% trifluoroacetic acid (TFA) in the presence of scavengers: 2.5% (*v*/*v*) H_2_O and 2.5% (*v*/*v*) triisopropylsilane (TIS) for 5 h. TFA was evaporated under a N_2_ flow and subsequently, diethyl ether was added to precipitate the crude peptides, which were next isolated by centrifugation (4000 rpm, 5 °C, 10 min). Precipitates were then re-dissolved in acetic acid 10% (*v*/*v*), frozen in a dry ice/acetone bath (−78 °C) and lyophilized. All peptides were purified by semi-preparative RP-HPLC in a Zorbax C-18 column (Agilent, 5 µm, 9.4 × 250 mm) at a flow rate of 2.5 mL/min and a detection wavelength of 220 nm. The solvents used as the eluting system were H_2_O (0.05% TFA) and CH_3_CN (0.05% TFA). A linear gradient of 5–95% of CH_3_CN in H_2_O over 20 min was performed after 5 min of isocratic elution at 5% CH_3_CN (0.05% TFA). After the purification of each peptide was completed, characterization was carried out by HPLC (Agilent Technologies 1260 Infinity, Santa Clara, CA, USA) and ESI-MS (Waters LCT Premier XE, Micromass Waters, Milford, MA, USA).

### 3.2. Home-Designed ELISA Assays

MaxiSorp microtiter plates were incubated with Neutravidin protein diluted in PBS (0.5 μg/well) overnight at 4 °C and thereafter for 1 h at 37 °C. After washing the plates, the biotinyl peptides were diluted at 1 μg/mL in PBS and 100 μL of the peptide solution was added to each well. The plates were incubated for 1 h at 37 °C. Subsequently, the plates were blocked with 2% BSA in PBS with 0.05% Tween-20 for 30 min at 37 °C. Then, the plates were washed 3 times. Sera were diluted 250-fold in RIA buffer (1% BSA *w/v*, 350 mM NaCl, 10 mM TRIS, 1% *v*/*v* Triton X-100, 0.5% *w/v* Na-deoxycholate, 0.1% *w/v* SDS) supplemented with 10% fetal bovine serum and 100 μL of the dilution was added to each well. The plates were incubated for 1 h at 37 °C and then overnight at 4 °C. Afterwards, each plate was washed 3 times with PBS/0.05% Tween-20 and 100 μL of anti-human IgG secondary antibody conjugated to peroxidase diluted in RIA buffer at 1:4000 was added to each well and incubated for 1 h at 37 °C. After washing the plates, detection of bound antibodies was carried out using SigmaFast (Sigma-Aldrich, St. Louis, MO, USA), with o-phenylenediamine dihydrochloride (OPD) as a substrate. Reaction was stopped with 50 μL of 2N H_2_SO_4_ and plates were read at 492 nm. Sera were from 172 RA patients diagnosed according to the 2010 ACR/EULAR criteria assessed in the Rheumatology Department of Hospital Clinic in Barcelona. Individuals fulfilling other inflammatory arthritis or connective tissue disease diagnostic criteria were excluded. All sera were tested in duplicate. A control healthy serum was included in each ELISA plate which showed no reactivity to any of the peptides. Results were obtained in values of optical density (OD) and ranged from 0 to 4, where 0 implies no reactivity and 4 is the highest value of intensity of reactivity. For easy comparison of the crude results, median values of the duplicates were calculated and were represented for better visualization in the form of a heat map [41].

### 3.3. Circular Dichroism Assays

Circular dichroism (CD) spectra were recorded on a Jasco J-1500 CD spectropolarimeter (Jasco Corporation, Tokyo, Japan) at 20 °C in a quartz cell with an optical length of 1 mm and in the region 190–260 nm. Peptides (0.5 mg/mL) were dissolved in distilled water, in carbonate buffer (pH = 10) or in the structure-promoting solvent trifluoroethanol (TFE). CD band intensities are expressed in molar ellipticity per residue. The mean residue ellipticity (MRE in deg cm^2^ dmol^−1^) was calculated using the equation θ_MRE_ = θ·Mr/c·l·n, where θ is the measured ellipticity, Mr the molecular weight of the peptide, c the concentration of the sample, l the pathlength of the cuvette and n the number of peptide bonds. The percentage of α-helix conformation in the peptides was estimated as described in [21] using the formalism of Chen et al. [22].

### 3.4. NMR Methodology

The 2D ^1^H-^1^H solution nuclear magnetic resonance (NMR) experiments were carried out to characterize peptides’ secondary structure. The samples were prepared by dissolving the lyophilized peptides at a concentration of 2 mM in a mixture of H_2_O/TFE-d_2_ (70:30 or 50:50, *v*/*v*). The pH was then adjusted to 3.0 using DCl/NaOD in D_2_O. Total correlation spectroscopy (^1^H-^1^H TOCSY), nuclear Overhauser effect spectroscopy (^1^H-^1^H NOESY) and heteronuclear single-quantum coherence (^1^H-^13^C HSQC) NMR experiments were performed at 25 °C on a Bruker Avance III HD spectrometer (Bruker BioSpin GmbH, Rheinstetten, Germany) operating at a ^1^H frequency of 500 MHz. A 5 mm triple-resonance (^1^H/^13^C/^15^N) gradient cryoprobe was used. Water suppression was achieved using excitation sculpting with gradients. The NMR spectra were processed using TopSpin 4.0.6 software (Bruker Biospin) and analyzed using CCPNmr Analysis 3.1.1 [42] for resonance assignment. NOE cross-peaks were integrated in 250 ms NOESY spectra, and the NOE volumes were converted into distances and calibrated using the average NOE volume of the resolved geminal methylene proton cross-peaks from His7. According to Wuthrich’s procedure [40], each distance was converted into a distance restraint by calculating the upper distance bounds and they were classified as strong (1.8–2.8 Å), medium (1.8–3.8 Å), weak (1.8–5.0 Å) or very weak. Pseudoatom corrections were applied to NOE constraints involving equivalent or non-stereo-assigned residues [39].

### 3.5. Molecular Dynamics

We ran molecular dynamics simulations using the PT-WTE enhanced sampling method [43]. Each peptide simulation followed the same protocol. An initial configuration was generated with Peptide Builder [44]. It was solvated in a box of 6.5 nm with NaCl. We used the Gromos 54a8 force field [45,46] with the post-translational modifications used in this work parameterized by the Zagrovich group [47] using the Viena-PTM web server [48]. The water model was simple point charge (SPC). The PT-WTE simulation used 24 replicas in the temperature range of 275 K to 500 K. The replica at 278.15 K (5 °C, matching NMR acquisition temperature) was not biased so that statistical properties could be obtained from it without the need for reweighting. The NMR NOEs were used as restraints in the Gromacs simulations. Each NOE strength was associated with a distance interval, summarized in Table 2. In case the hydrogen atom associated with said NOE was not available, the bound carbon atom was used for reference and an additional 1.1 Å were added to take into account the typical C-H bond distance. An averaged force of 1000 kJ mol^−1^ nm^−2^ was applied to maintain the specified restraints. The potential form for distance restraints was quadratic below a specified lower bound (R0) and between two specified upper bounds (R0 and R1), and linear beyond the largest bound (R2). The R0 and R1 distances were derived from the minimum and maximum values obtained from the NOE data, which are presented in Tables S2–S4. The R2 distance was calculated as a 50% increase from the R1 distance, except in the case of “very weak” restraints. For the latter, a distance of 10 nm was chosen for both R1 and R2, resulting in an essentially unbounded upper limit for the restraint, as the distance between two residues in such a short peptide will never exceed 10 nm. Peptides P1, P2 and P10 had 54, 66 and 50 restraints, respectively. The restraints were applied to all the distances of all the replicas. Each peptide was simulated for 200 ns and the first 40 ns were discarded for the statistical analysis as an equilibration phase. Chemical shifts were back-calculated for the non-modified residues with Sparta+ [49]. The reported values corresponded to the average of 500 frames obtained from the equilibrated region of each peptide trajectory.

The three peptide trajectories are analyzed with an in-house-developed clustering algorithm based on the article published by Appadurai and coworkers [50] that will be described in detail elsewhere. Briefly, the algorithm generates a pairwise similarity matrix of all the frames in the three trajectories. The structural similarity matrix is then reduced to a 2D representation using T-SNE, and points that are sufficiently close together are mapped into the same cluster. All the molecular dynamics are performed with Gromacs 2022.5 [51,52] patched with the open-source community-developed PLUMED library [53] version 2.9.0 [54]. Analysis of the results uses numpy [55] and the data representation uses MatPlotLib [56].

## 4. Conclusions

The overall analysis of these simulations, including interatomic distance restraints arising from NMR, reveals that the three peptides adopt distinct conformations, and that secondary structure content is not sufficient to explain the difference between P1 and P2, which mainly arises from more compact configurations in P2. These different configurations, likely resulting from the lower net charge of P2 relative to P1, may explain their autoantibody recognition, as they are the same as in P10. The effect of the PTMs in P2 and P10 results in similar intra-molecular contacts both around residue 5 and the C-terminus. Consistent with this, we found some cross-reactivity in patient samples. However, we also observed the presence of discordant sera, which showed different reactivity toward P2 and P10, which may be in accordance not only with the presence of different PTMs but also with the different overall 3D structure described for these two peptides. In summary, differences in autoantibody reactivity between peptides bearing different PTMs within their primary structures are noted. Peptides with PTMs adopt different conformations than the unmodified peptide, probably due to the lower net charge of peptides with multiple PTMs, which may explain their recognition by autoantibodies.

## Figures and Tables

**Table 1:** Primary peptide sequence and characterization by analytical HPLC and ESI-MS.

Peptide	Sequence	RP-HPLC	ESI-MS	
		R_t_ (min)	Calculated	Experimental
P1 (control)	Biotinyl-PEG_2_-HSTKRGHAKSRPVRG	12.1	2216.6	2215.8
P2	Biotinyl-PEG_2_-HSTKCitGHAKSRPVCitG	11.9	2218.6	2217.5
P3	Biotinyl-PEG_2_-HSThCitRGHAhCitSRPVRG	11.9	2302.6	2301.5
P4	Biotinyl-PEG_2_-HSTK(Ac)RGHAK(Ac)SRPVRG	12.2	2300.7	2299.5
P5	Biotinyl-PEG_2_-HSThCitCitGHAhCitSRPVCitG	12.3	2304.6	2303.5
P6	Biotinyl-PEG_2_-HSTK(Ac)CitGHAK(Ac)SRPVCitG	12.5	2302.6	2301.6
P7	Biotinyl-PEG_2_-HSTK(Ac)RGHAhCitSRPVRG	12.1	2301.6	2300.4
P8	Biotinyl-PEG_2_-HSThCitRGHAK(Ac)SRPVRG	11.8	2301.6	2300.6
P9	Biotinyl-PEG_2_-HSTK(Ac)CitGHAhCitSRPVCitG	12.2	2303.6	2302.6
P10	Biotinyl-PEG_2_-HSThCitCitGHAK(Ac)SRPVCitG	12.3	2303.6	2302.7
P11	Biotinyl-PEG_2_-HSTK(Ac)RGHAhCitSRPVCitG	12.1	2302.6	2301.6

Cit: citrulline; hCit: homocitrulline; K(Ac): acetyl-lysine

**Table 2:** Distance intervals associated with restraint strength.

Restraint Strength	R0 Distance (Å)	R1 Distance (Å)	R2 Distance (Å)
Very weak	0.55	10	10
Weak	0.18	0.55	0.83
Medium	0.18	0.33	0.5
Strong	0.18	0.25	0.38

## Data Availability

Data are contained within the article or Supplementary Materials. The data presented in this study are available on request from the corresponding author.

## Supplementary Materials

The following supporting information can be downloaded at https://www.mdpi.com/article/10.3390/ijms26189026/s1.

## Abbreviations

The following abbreviations are used in this manuscript:

RA	Rheumatoid arthritis
PTMs	Post-translational modifications
PAD	Peptidylarginine deiminase enzyme
AMPAs	Anti-modified protein/peptide antibodies
AF2	AlphaFold 2
MHC	Major histocompatibility complex
NMR	Nuclear magnetic resonance
TFE	Trifluoroethanol
pLDDT	Predicted local distance difference test
PEG	Poly(ethylene glycol)
SPPS	Solid-phase peptide synthesis
HPLC	High-performance liquid chromatography
ESI-MS	Electrospray mass spectrometry
Cit	Citrulline
hCit	Homocitrulline
KAc	Acetyl-lysine
OD	Optical density
CD	Circular dichroism
NOESY	Nuclear Overhauser effect spectroscopy
TOCSY	Total correlation spectroscopy
HSQC	Heteronuclear single-quantum coherence
MD	Molecular dynamics
Rg	Radius of gyration
Fmoc	9-Fluorenyl-methoxycarbonyl
Trt	Triphenylmethyl
tBu	Tert-butyl
Pmc	2,2,5,7,8-Pentamethyl-chroman-6-sulfonyl
HATU	2-(1H-7-azabenzotriazole-1-yl)-1,1,3,3-tetramethyluronium hexafluorophosphate methanaminium
DIPEA	*N,N*-Diisopropylethylamine
DMF	*N,N*-Dimethylformamide
PyBOP	Benzotriazole-1-yloxytris(pirrolidino) phosphonium hexafluorophosphate
HOBt	1-Hydroxybenzotriazole
TFA	Trifuoroacetic acid
TIS	Triisopropylsilane
ELISA	Enzyme-linked immunosorbent assay
PBS	Phosphate-buffered saline
BSA	Bovine serum albumin
TRIS	Tris(hydroxymethyl)aminomethane
SDS	Sodium dodecyl sulfate
SPC	Simple point charge
