# Supplementary material for: Structural Study of a Peptide Epitope Bearing Multiple Post-Translational Modifications in Rheumatoid Arthritis
Source: Int J Mol Sci. 2025 Sep 16;26(18):9026. doi: 10.3390/ijms26189026 (PMC12469451; doi:10.3390/ijms26189026)
Supplement: Supplementary file 1 [file ijms-26-09026-s001.zip › ijms-3780375-supplementary.pdf]

Structural Study of a Peptide Epitope Bearing Multiple Post-Translational Modifications in Rheumatoid Arthritis

María José Gómara [1,*], Cristina García-Moreno [1], Oriol Bárcenas [2,3], Raúl Castellanos-Moreira [4], Juan Camilo Sarmiento [4], Ramon Crehuet [2], Yolanda Pérez [5], Raimon Sanmartí [4] and Isabel Haro [1,*]

1 Unit of Synthesis and Biomedical Applications of Peptides, IQAC-CSIC, Jordi Girona 18-26, 08034 Barcelona, Spain
2 Computational and Theoretical Chemistry Group, IQAC-CSIC, Jordi Girona 18-26, 08034 Barcelona, Spain; oriol.barcenas@iqac.csic.es (O.B.); ramon.crehuet@iqac.csic.es (R.C.)
3 Institut de Biotecnologia i de Biomedicina and Departament de Bioquímica i Biologia Molecular, Universitat Autònoma de Barcelona, 08193 Barcelona, Spain
4 Department of Rheumatology, Hospital Clínic of Barcelona, 08036 Barcelona, Spain; sarmiento@clinic.cat (J.C.S.); sanmarti@clinic.cat (R.S.)
5 NMR Facility, IQAC-CSIC, Jordi Girona 18-26, 08034 Barcelona, Spain; yolanda.perez@iqac.csic.es
* Correspondence: mariajose.gomara@iqac.csic.es (M.J.G.); isabel.haro@iqac.csic.es (I.H.)

Supplementary Material

Figure S1. Analytical High Performance Liquid Chromatography (HPLC) and Electrospray Mass Spectrometry (ESI-MS) of P1-P11 peptides

Figure S2. CD Spectra of P1-P11 peptides in water. In the legend are indicated the peptides net charge at neutral pH.

Figure S3. The aliphatic and amide regions of the P2 peptide ^{1}H NMR spectra.

Figure S4. The aliphatic and amide regions of the P10 peptide ^{1}H NMR spectra.

Figure S5. The aliphatic and amide regions of the P1 peptide ^{1}H NMR spectra.

Figure S6. Comparison of the amide region of the ^{1}H NMR spectra of the P1, P2 and P10 peptides in a 70:30 (v/v) solution of H_2O/TFE-d2.

Figure S7. (Pages 19 and 20) Selected regions of the 2D NOESY spectrum of P1 peptide in a 70:30 (v/v) solution of H_2O/TFE-d2 at pH 3 (500 MHz, 298 K). The regions are displayed at different levels to make the lower-intensity correlation peaks more visible.

Figure S8. (Pages 21 and 22) Selected regions of the 2D NOESY spectrum of P2 peptide in a 70:30 (v/v) solution of H_2O/TFE-d2 at pH 3 (500 MHz, 298 K). The regions are displayed at different levels to make the lower-intensity correlation peaks more visible.

Figure S9. (Pages 23 and 24) Selected regions of the 2D NOESY spectrum of P10 peptide in a 70:30 (v/v) solution of H_2O/TFE-d2 at pH 3 (500 MHz, 298 K). The regions are displayed at different levels to make the lower-intensity correlation peaks more visible.

Figure S10. Chemical Shift (CS) difference between the experimental (NMR) and calculated CS from the peptide trajectories for the HA and the CA atoms. Sparta+ can only calculate the CS of residues without post-translational modifications, which is why some CS are missing.

Figure S11. Intramolecular contacts arising from the Molecular Dynamics trajectories. The i,i+3 and i,i+4 contacts are typical of α-helices and more prevalent in P10. P2 shows more contacts than P1 because it is more compact, especially in the C-term region. This region is similar to P10 region, as expected because they share the same sequence.

Table S1. Peptides net charge and estimation of the α-helix content according to molar ellipticity at 222nm.

Table S2. P1 peptide NOE restraints in 30% TFE.

Table S3. P2 peptide NOE restraints in 30% TFE.

Table S4. P10 peptide NOE restraints in 30% TFE.

P1

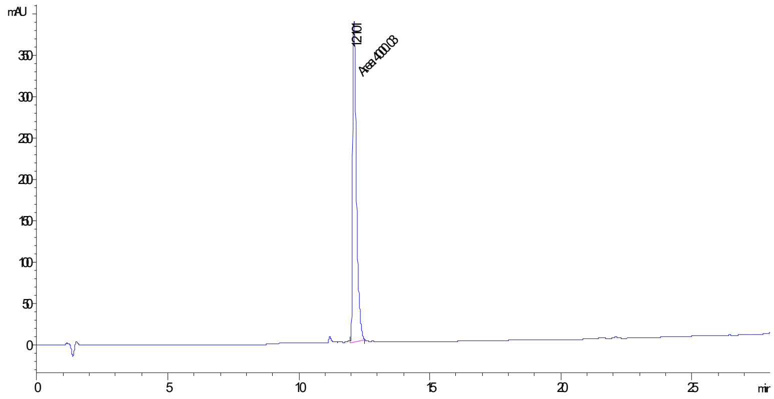

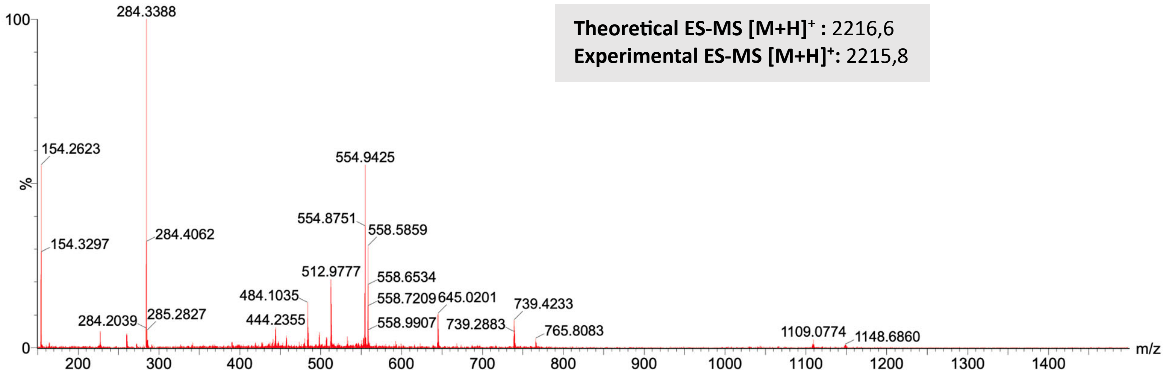
Theoretical ES-MS [M+H]+ : 2216,6
Experimental ES-MS [M+H]+: 2215,8
284.3388
154.2623
554.9425
554.8751
558.5859
284.4062
154.3297
512.9777
558.6534
484.1035
558.7209
645.0201
739.4233
285.2827
284.2039
444.2355
558.9907
739.2883
765.8083
1109.0774
1148.6860
%
m/z

P2

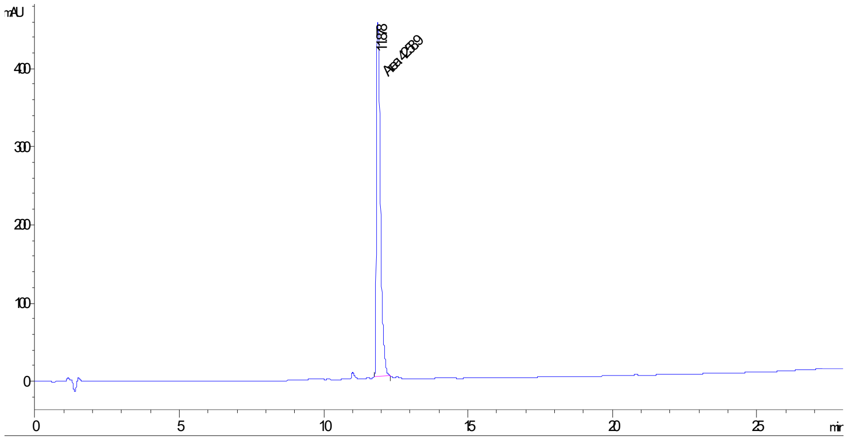
mAU
400
300
200
100
0
0
5
10
15
20
25
min

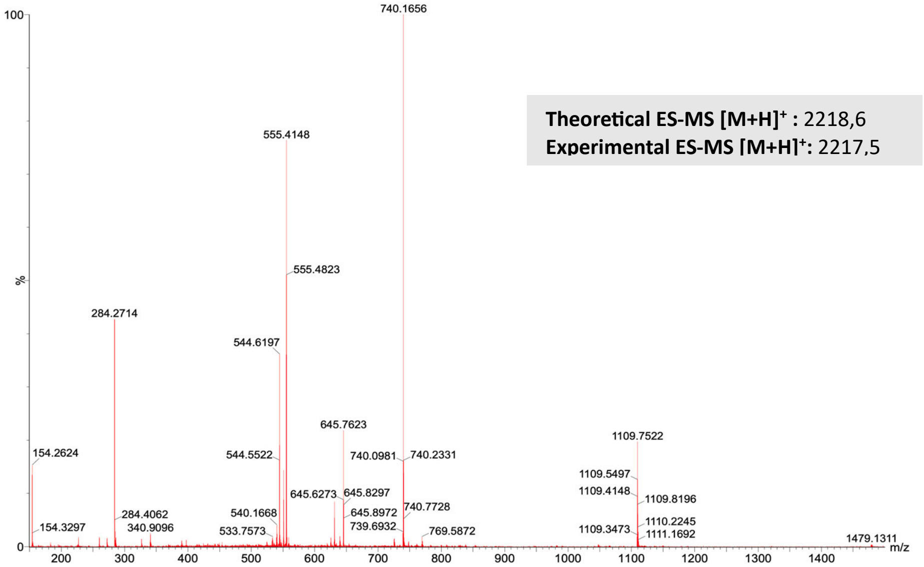
Theoretical ES-MS [M+H]+ : 2218,6
Experimental ES-MS [M+H]+: 2217,5
100
%
0
740.1656
555.4148
555.4823
284.2714
544.6197
645.7623
1109.7522
154.2624
544.5522
740.0981
740.2331
1109.5497
1109.4148
1109.8196
645.6273
645.8297
740.7728
540.1668
645.8972
284.4062
154.3297
340.9096
533.7573
739.6932
1109.3473
1110.2245
769.5872
1111.1692
1479.1311
m/z
200
300
400
500
600
700
800
900
1000
1100
1200
1300
1400

P3

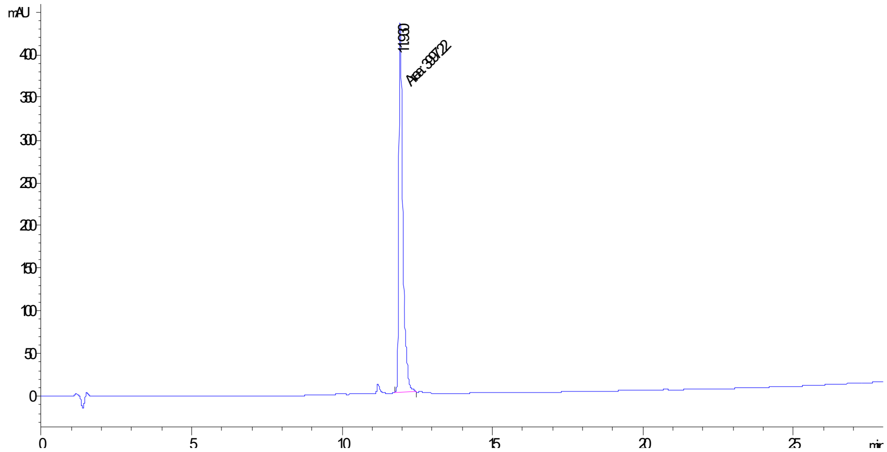

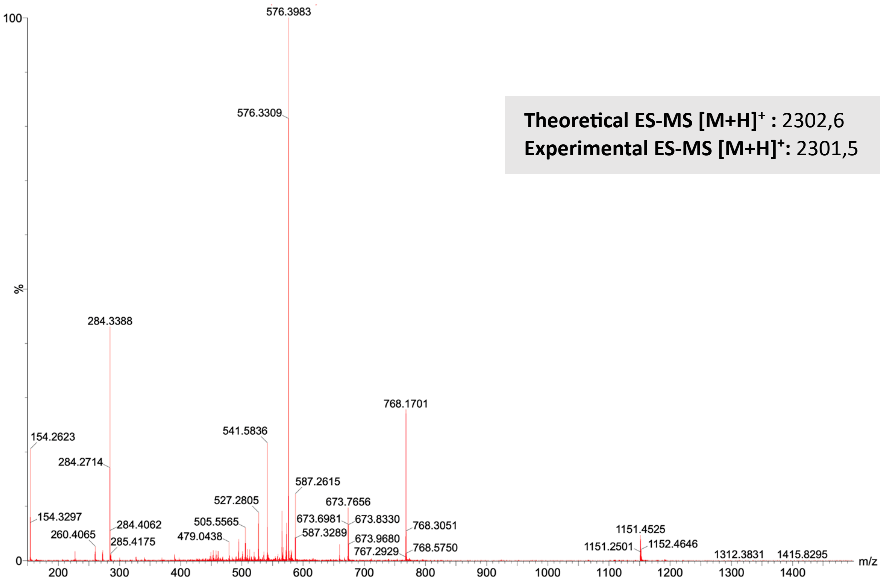

Theoretical ES-MS $[M+H]^+$: 2302,6
Experimental ES-MS $[M+H]^+$: 2301,5

P4

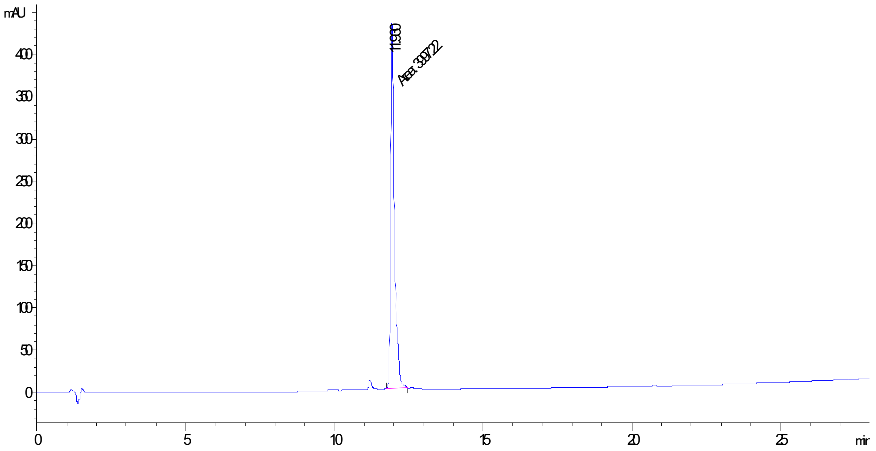
mAU
min

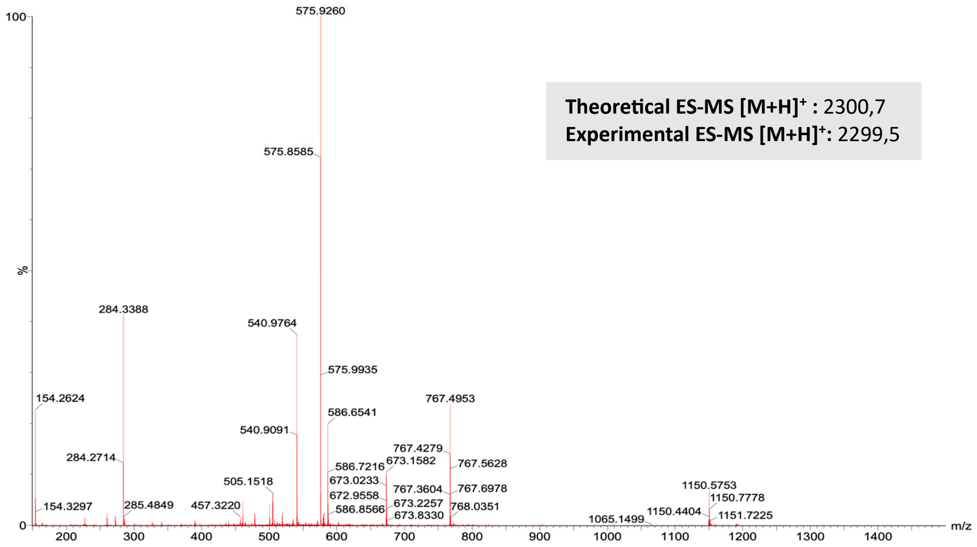
575.9260
575.8585
284.3388
540.9764
575.9935
154.2624
767.4953
586.6541
540.9091
767.4279
284.2714
586.7216
673.1582
767.5628
505.1518
673.0233
672.9558
767.3604
767.6978
154.3297
285.4849
457.3220
673.2257
586.8566
673.8330
768.0351
1150.5753
1150.7778
1150.4404
1065.1499
1151.7225
m/z
%
Theoretical ES-MS [M+H]$^+$: 2300,7
Experimental ES-MS [M+H]$^+$: 2299,5

P5

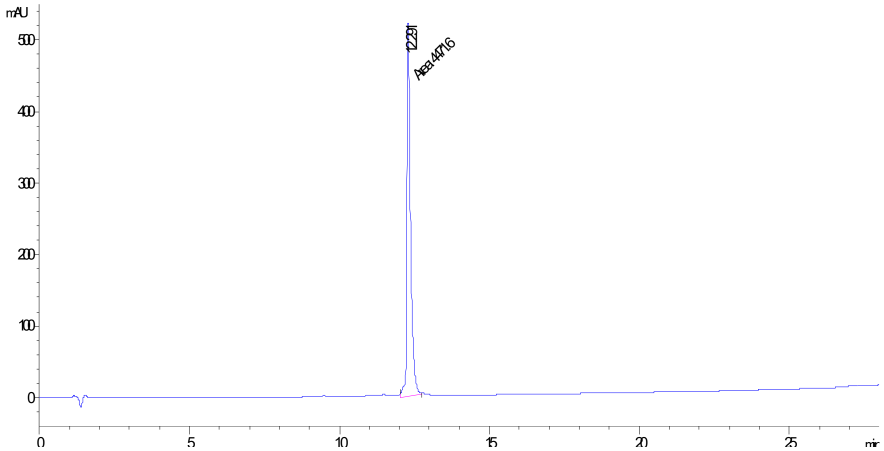
mAU
500
400
300
200
100
0
0
5
10
15
20
25

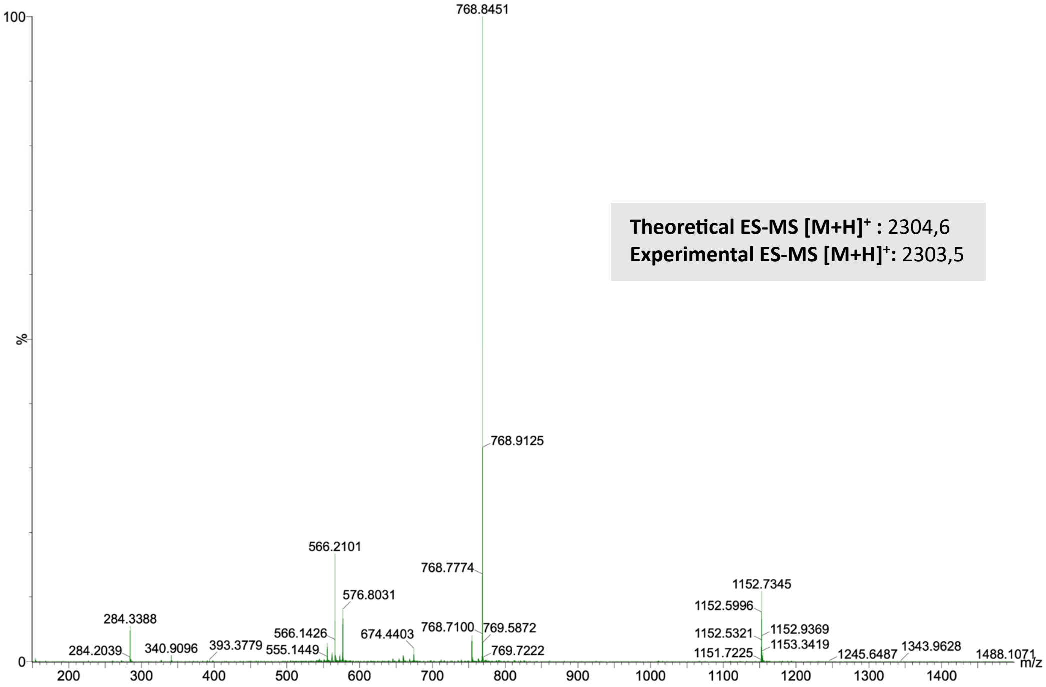
768.8451
768.9125
566.2101
768.7774
576.8031
1152.7345
284.3388
1152.5996
566.1426
674.4403
768.7100
769.5872
1152.5321
1152.9369
284.2039
340.9096
393.3779
555.1449
769.7222
1151.7225
1153.3419
1245.6487
1343.9628
1488.1071
100
%
0
200
300
400
500
600
700
800
900
1000
1100
1200
1300
1400
m/z
Theoretical ES-MS [M+H]$^+$: 2304,6
Experimental ES-MS [M+H]$^+$: 2303,5

P6

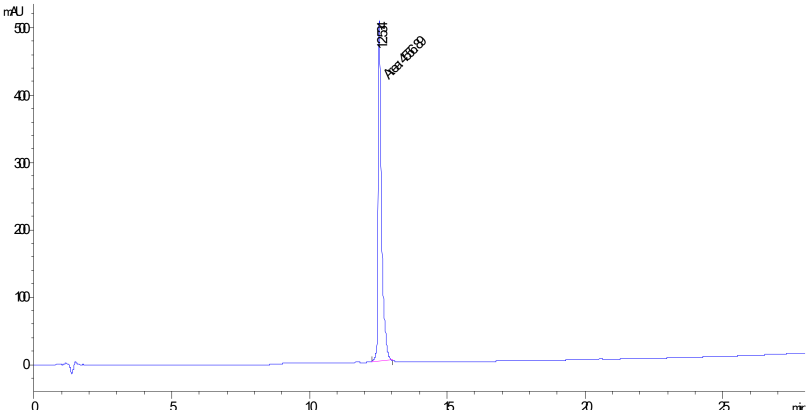
mAU
500
400
300
200
100
0
0
5
10
15
20
25

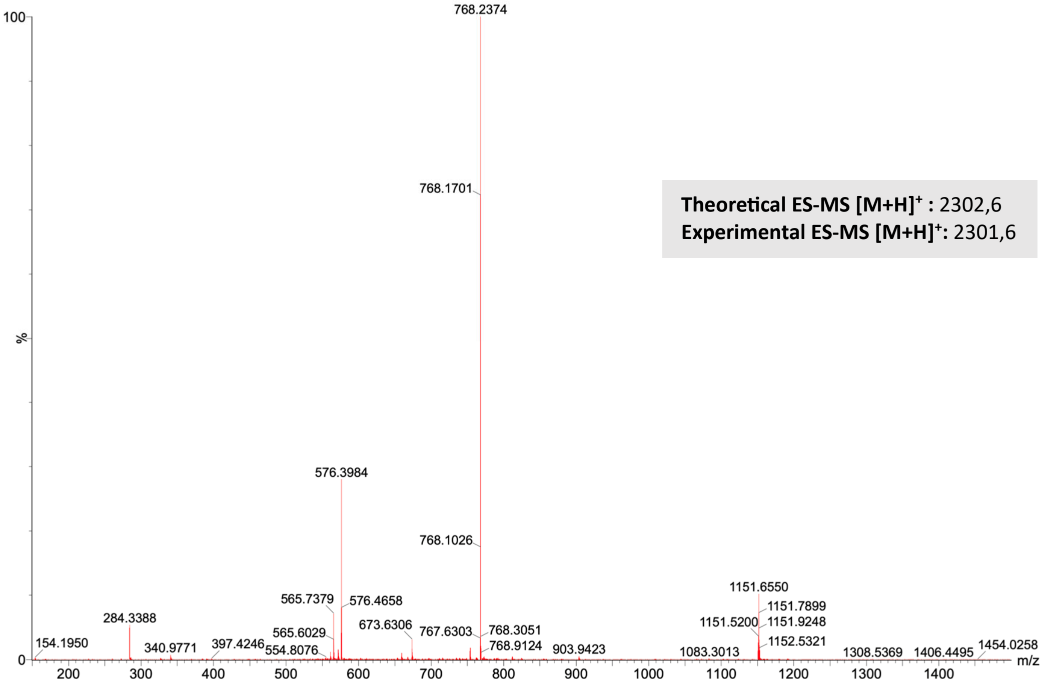
100
%
0
768.2374
768.1701
576.3984
768.1026
1151.6550
1151.7899
1151.9248
1151.5200
1152.5321
565.7379
576.4658
284.3388
565.6029
673.6306
767.6303
768.3051
768.9124
154.1950
340.9771
397.4246
554.8076
903.9423
1083.3013
1308.5369
1406.4495
1454.0258
200
300
400
500
600
700
800
900
1000
1100
1200
1300
1400
m/z
Theoretical ES-MS [M+H]$^+$: 2302,6
Experimental ES-MS [M+H]$^+$: 2301,6

P7

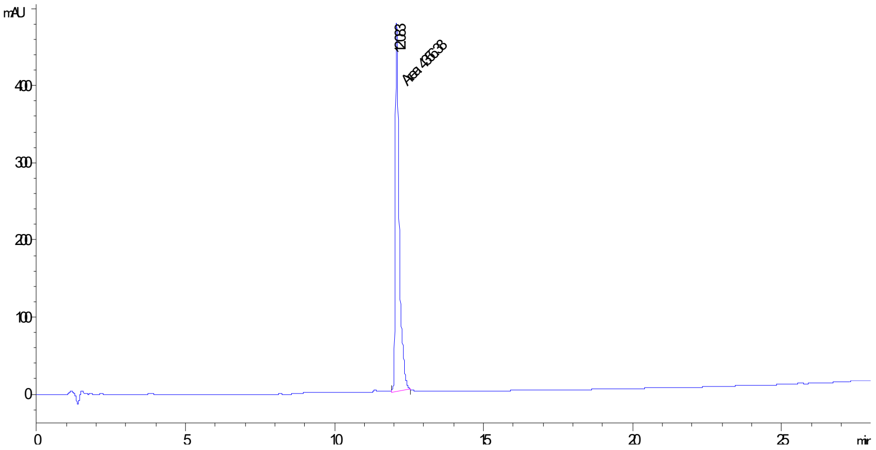

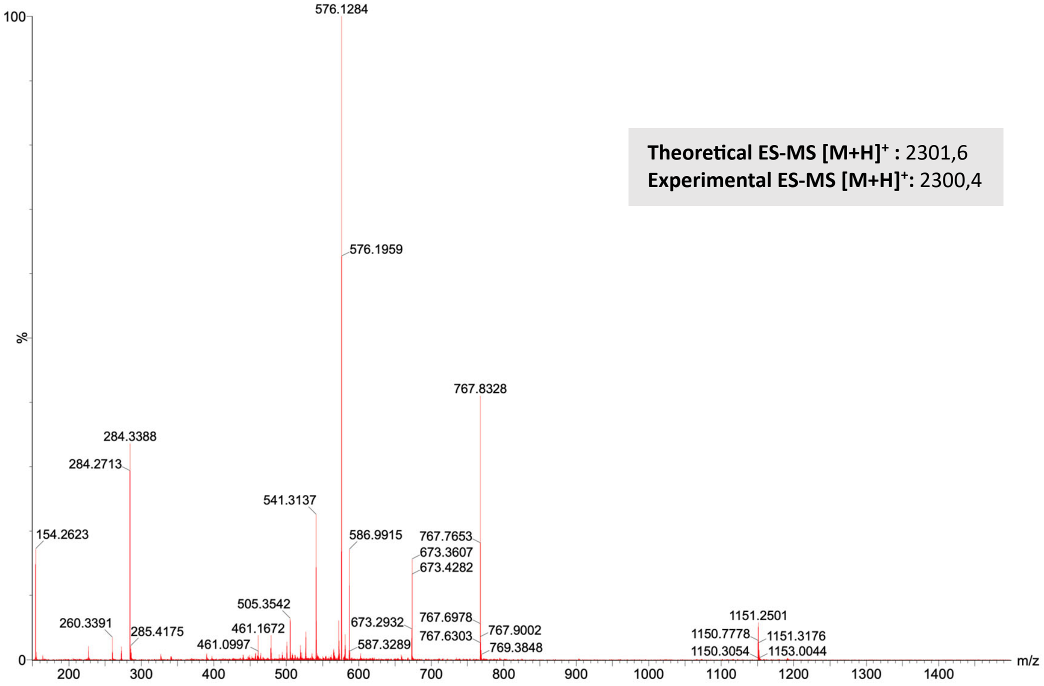

Theoretical ES-MS [M+H]$^{+}$: 2301,6
Experimental ES-MS [M+H]$^{+}$: 2300,4

P8

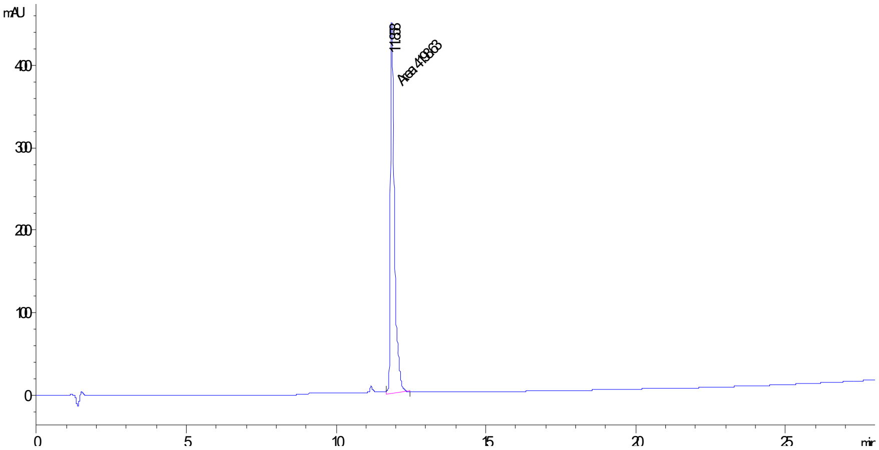

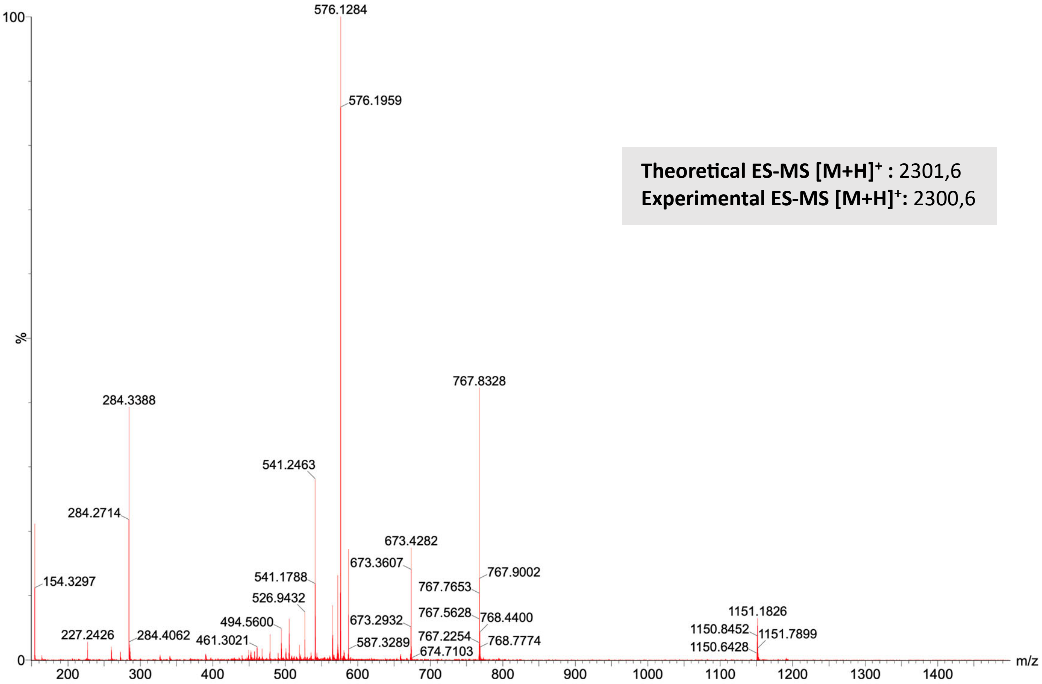

Theoretical ES-MS [M+H]$^+$: 2301,6
Experimental ES-MS [M+H]$^+$: 2300,6

P9

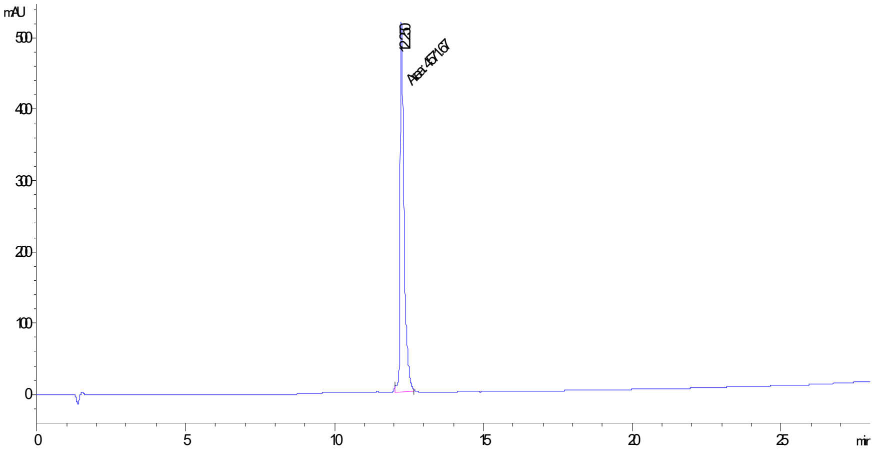
mAU
500
400
300
200
100
0
0
5
10
15
20
25
min

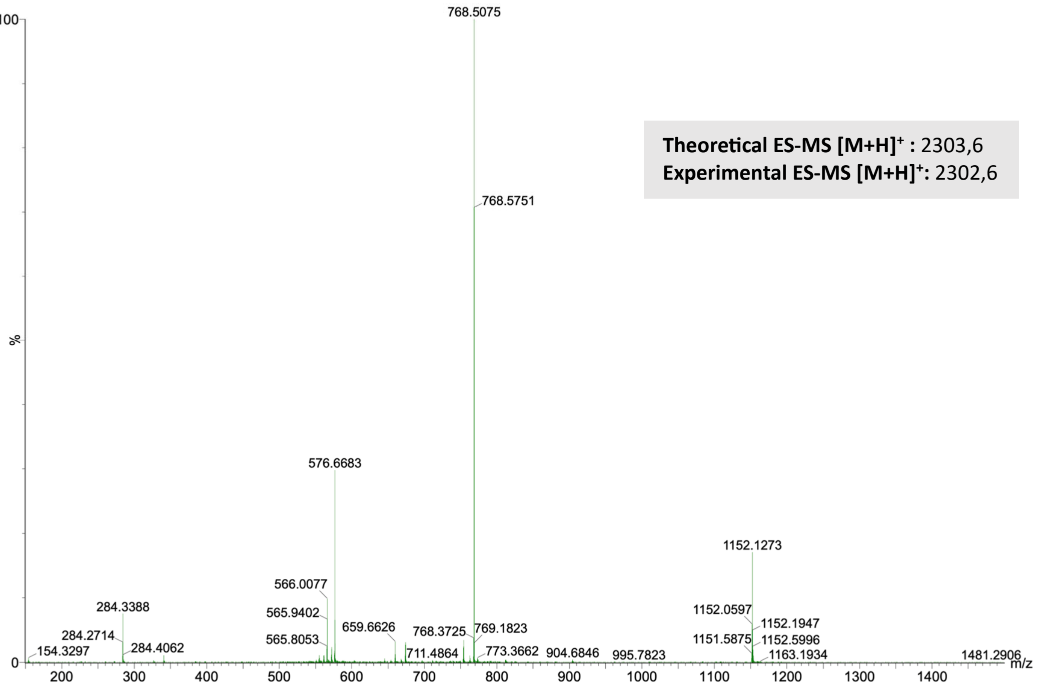
100
%
0
768.5075
768.5751
576.6683
1152.1273
566.0077
565.9402
565.8053
284.3388
284.2714
284.4062
154.3297
659.6626
768.3725
769.1823
711.4864
773.3662
904.6846
995.7823
1152.0597
1152.1947
1151.5875
1152.5996
1163.1934
1481.2906
200
300
400
500
600
700
800
900
1000
1100
1200
1300
1400
m/z
Theoretical ES-MS [M+H]+ : 2303,6
Experimental ES-MS [M+H]+: 2302,6

P10

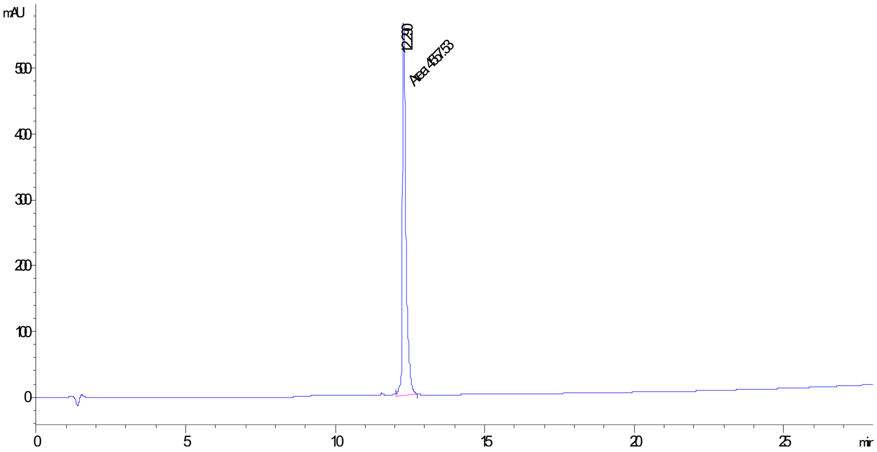

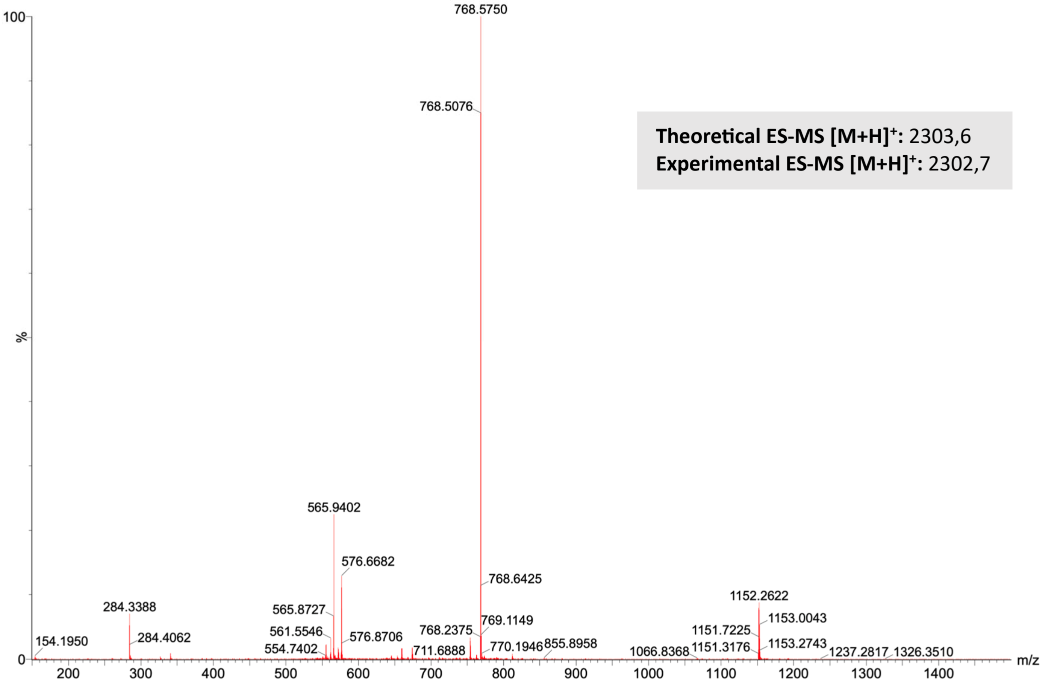

Theoretical ES-MS [M+H]$^{+}$: 2303,6
Experimental ES-MS [M+H]$^{+}$: 2302,7

P11

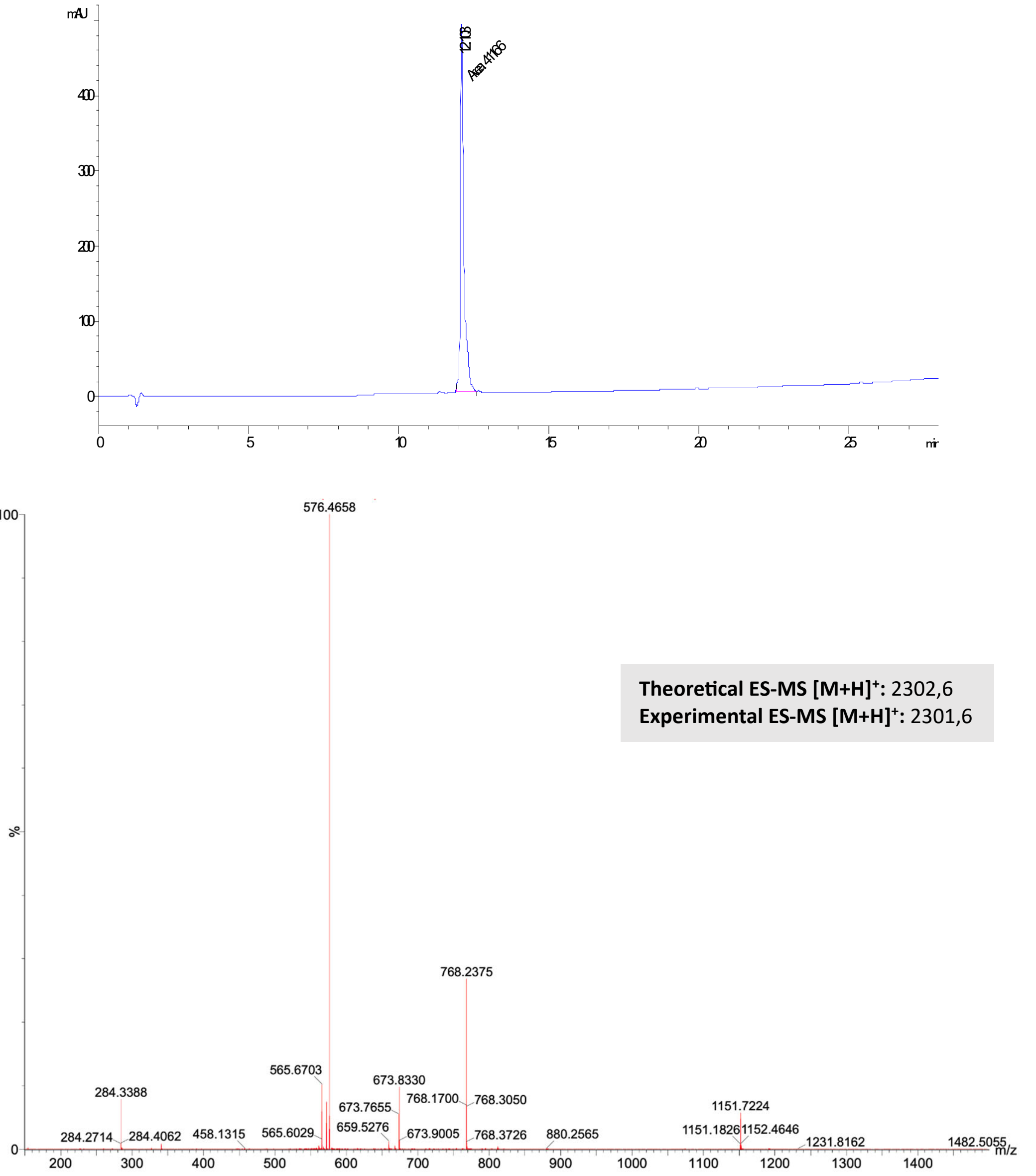

Figure S1. Analytical High Performance Liquid Chromatography (HPLC) and Electrospray Mass Spectrometry (ESI-MS) of P1-P11 peptides

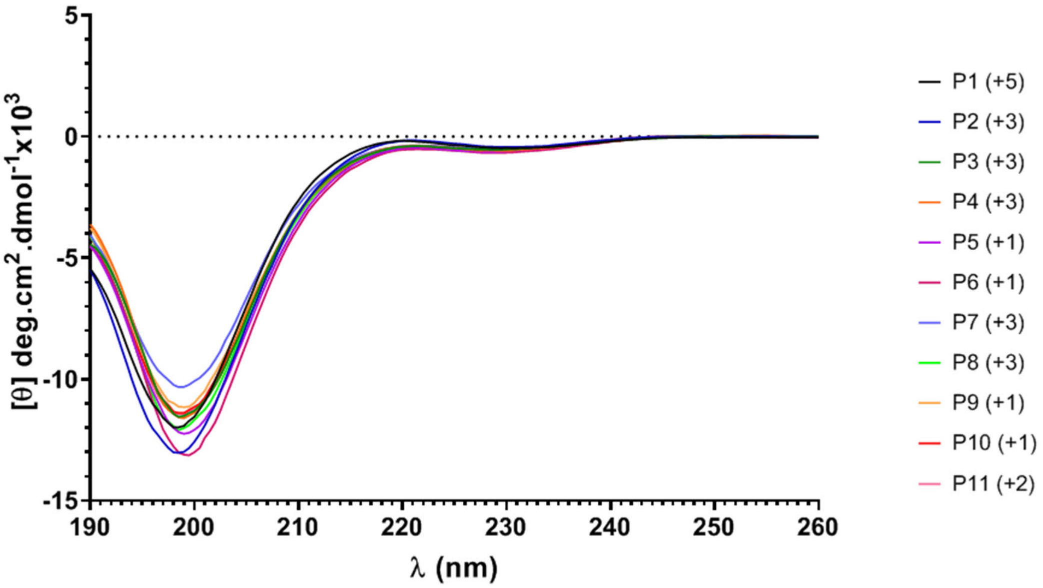

Figure S2. CD Spectra of P1-P11 peptides in water. In the legend are indicated the peptides net charge at neutral pH.

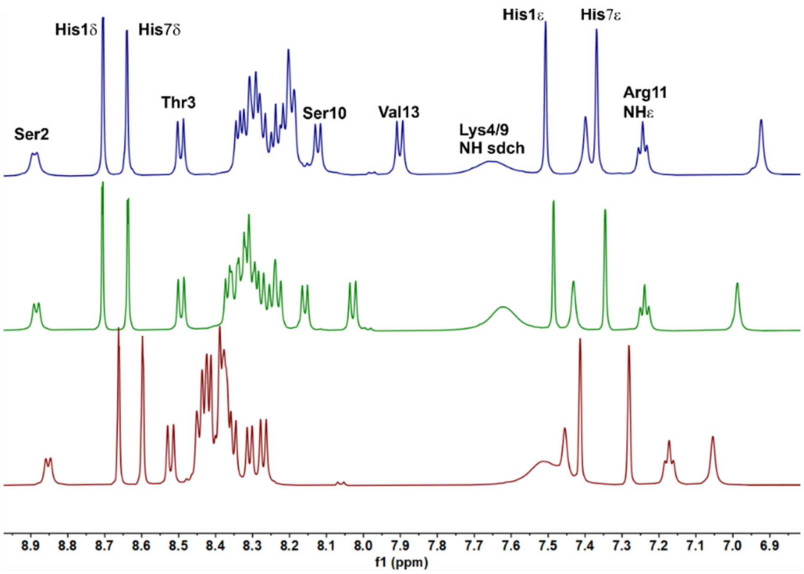

Figure S3. The aliphatic and amide regions of the P2 peptide ^{1}H NMR spectra in 90:10 H_2O/D_2O, 70:30 and 50:50 v:v H_2O/TFE-d2 (pH = 3, 500 MHz, 298 K) are shown in red, green and blue, respectively.

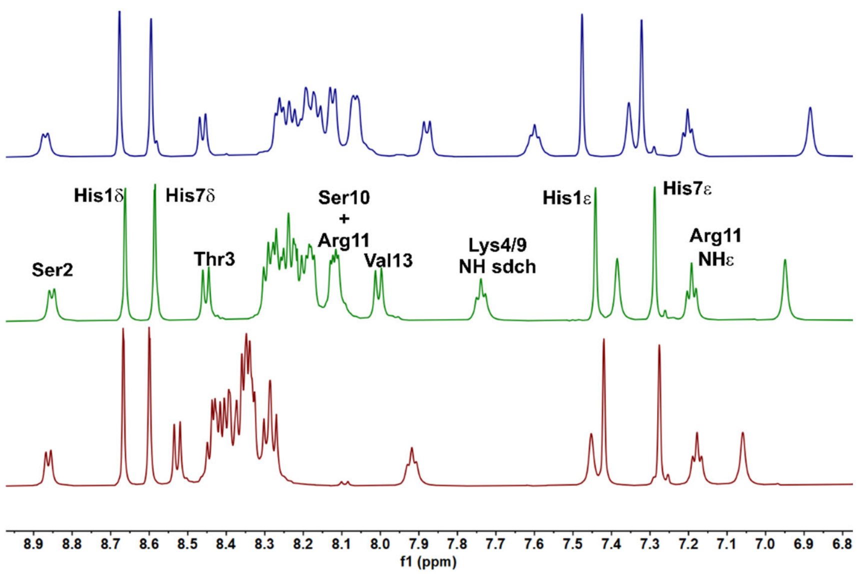

Figure S4. The aliphatic and amide regions of the P10 peptide ^{1}H NMR spectra in 90:10 H_2O/D_2O, 70:30 and 50:50 (v:v) H_2O/TFE-d_2 (pH = 3, 500 MHz, 298 K) are shown in red, green and blue, respectively.

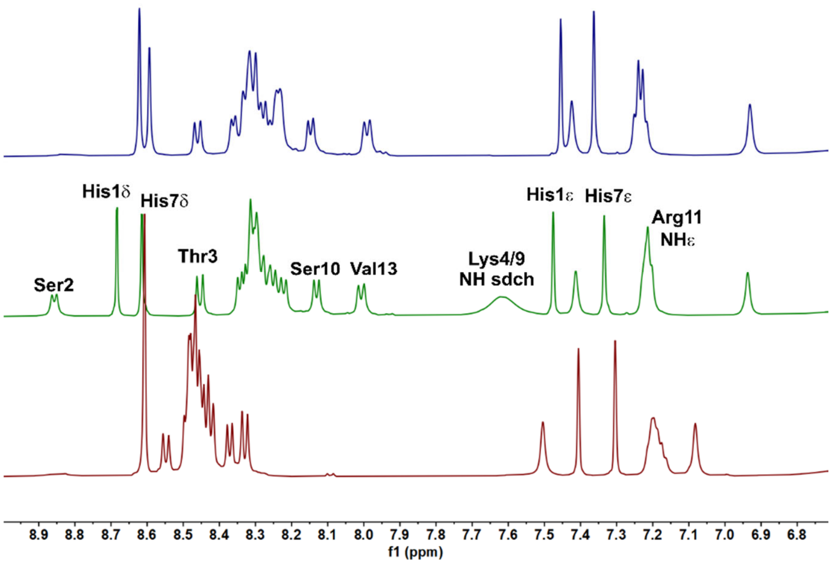

Figure S5. The aliphatic and amide regions of the P1 peptide ^1H NMR spectra in 90:10 H_2O/D_2O, 70:30 and 50:50 (v/v) H_2O/TFE-d_2 (pH = 3, 500 MHz, 298 K) are shown in red, green and blue, respectively.

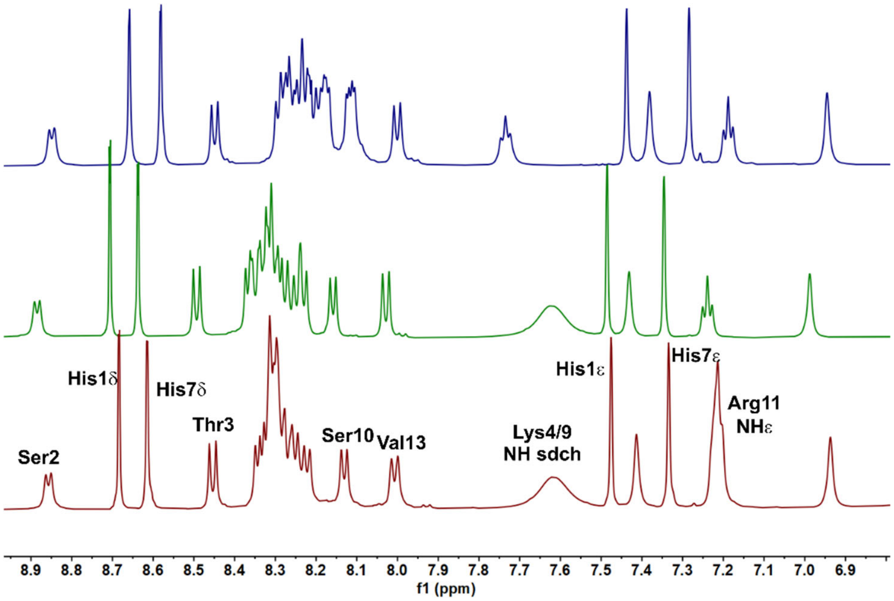

Figure S6. A comparison of the amide region of the ^{1}H NMR spectra of the P1 (red), P2 (green) and P10 (blue) peptides in a 70:30 (v/v) solution of H_2O/TFE-d2 at pH 3 (500 MHz, 298 K).

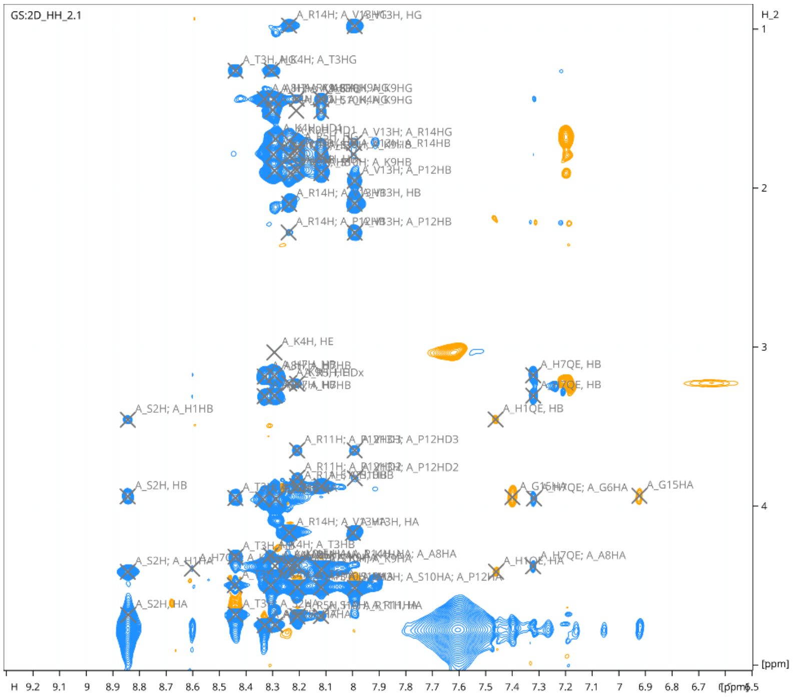
GS:2D_HH_2.1
H_2
A_T3H, HG
A_V13H; A_R14HG
A_V13H; A_R14HB
A_V13H; A_P12HB
A_K4H, HE
A_H7QE, HB
A_S2H; A_H1HB
A_H1QE, HB
A_S2H, HB
A_G15HA
A_R14H; A_V13H, HA
A_H7QE; A_A8HA
A_S2H, HA
[ppm]

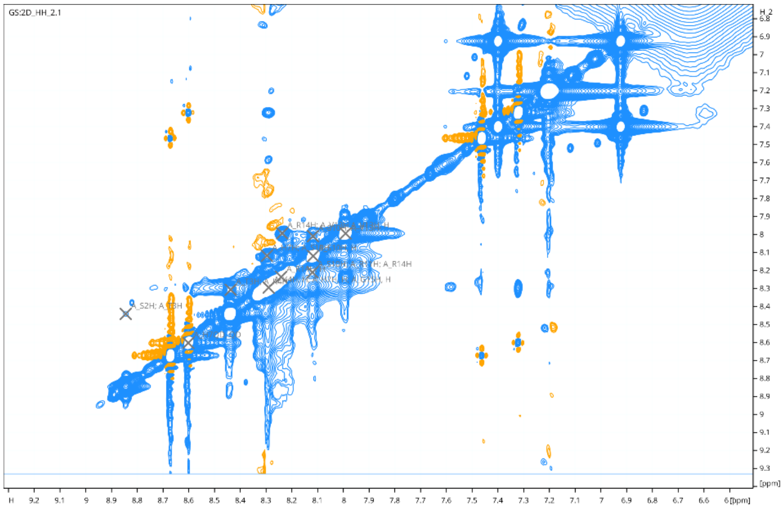
GS:2D_HH_2.1
H_2
[ppm]

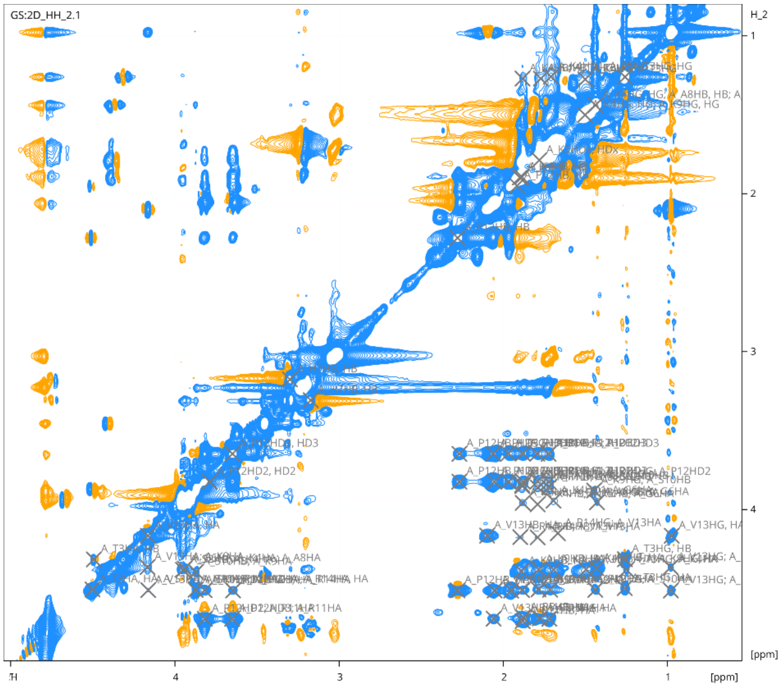

Figure S7. (Pages 19 and 20) Selected regions of the 2D NOESY spectrum of P1 peptide in a 70:30 (v/v) solution of H_2O/TFE-d2 at pH 3 (500 MHz, 298 K). The regions are displayed at different levels to make the lower-intensity correlation peaks more visible.

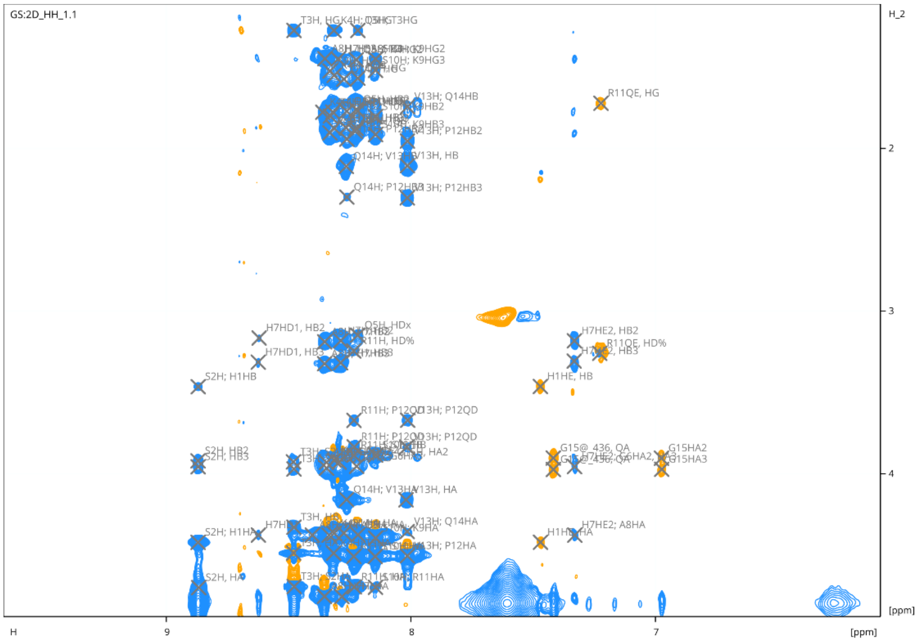
GS:2D_HH_1.1
H_2
H
[ppm]
R11QE, HG
V13H; Q14HB
Q14H; V13H, HB
V13H; P12HB2
Q14H; P12HB3
V13H; P12HB3
H7HD1, HB2
H7HD1, HB3
H7HE2, HB2
R11QE, HD%
R11H, HD%
S2H; H1HB
H1HE, HB
R11H; P12QD
V13H; P12QD
S2H; HB2
S2H; HB3
G15HA2
G15HA3
Q14H; V13HA
V13H, HA
V13H; Q14HA
H7HE2; A8HA
S2H; H1HA
V13H; P12HA
S2H, HA

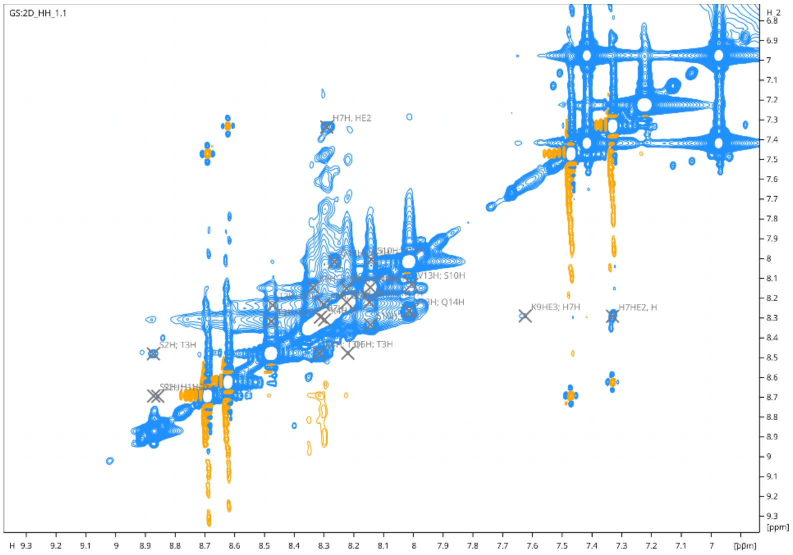
GS:2D_HH_1.1
H_2
H
[ppm]
H7H, HE2
V13H; S10H
K9HE3; H7H
H7HE2, H
S2H; T3H

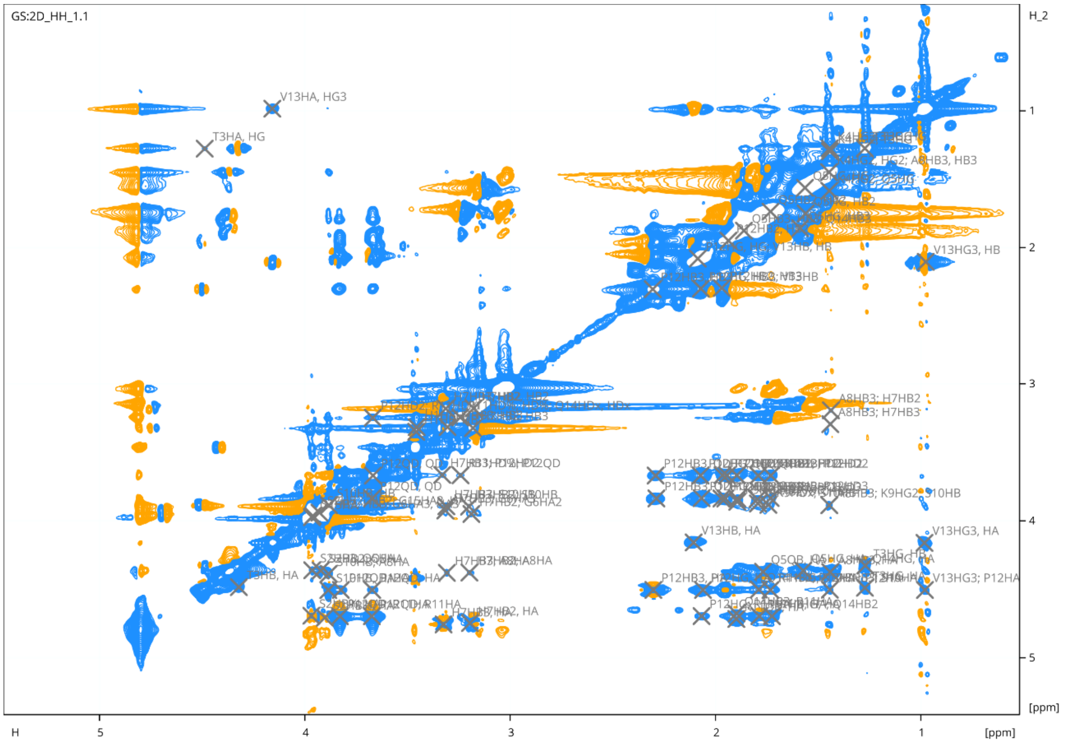

Figure S8. (Pages 21 and 22) Selected regions of the 2D NOESY spectrum of P2 peptide in a 70:30 (v/v) solution of H_2O/TFE-d2 at pH 3 (500 MHz, 298 K). The regions are displayed at different levels to make the lower-intensity correlation peaks more visible.

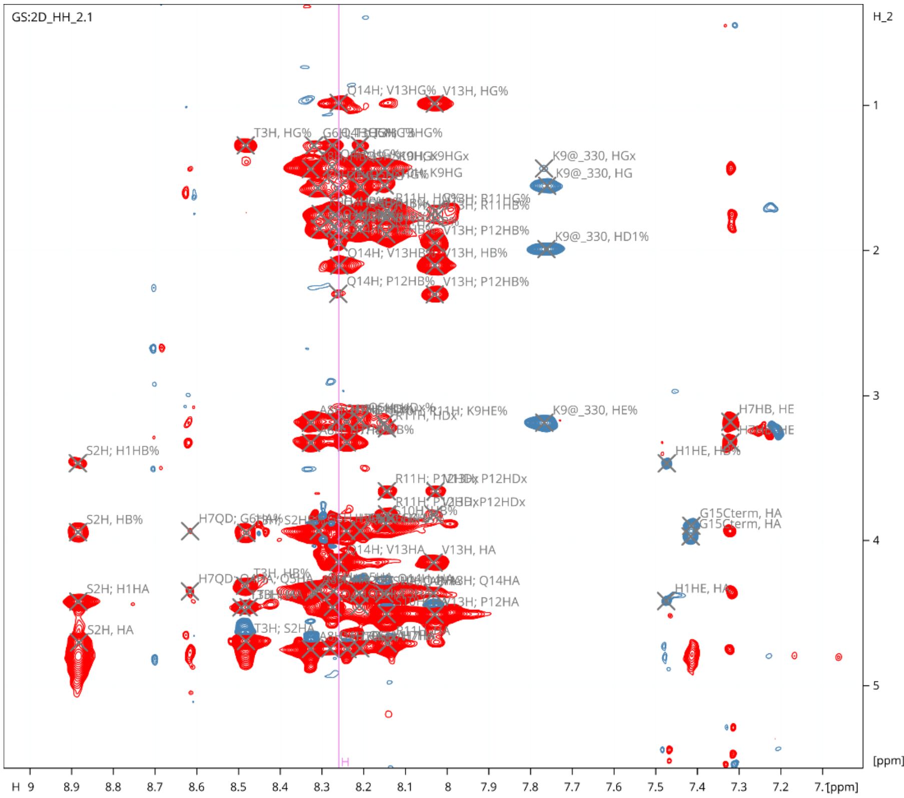
GS:2D_HH_2.1
H_2
Q14H; V13HG%
V13H, HG%
T3H, HG%
K9@_330, HGx
K9@_330, HG
K9@_330, HD1%
V13H; P12HB%
Q14H; P12HB%
K9@_330, HE%
H7HB, HE
S2H; H1HB%
S2H, HB%
H7QD; G6HA%
G15Cterm, HA
Q14H; V13HA
V13H, HA
S2H; H1HA
H1HE, HA
V13H; P12HA
S2H, HA
T3H; S2HA
[ppm]
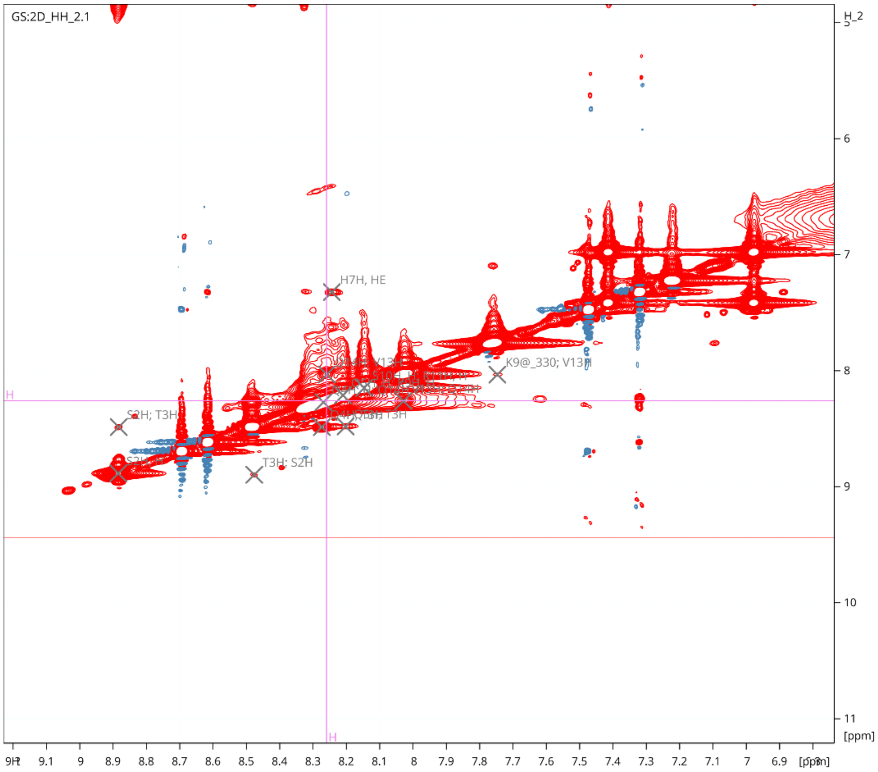
GS:2D_HH_2.1
H_2
H7H, HE
K9@_330; V13H
S2H; T3H
T3H; S2H
[ppm]

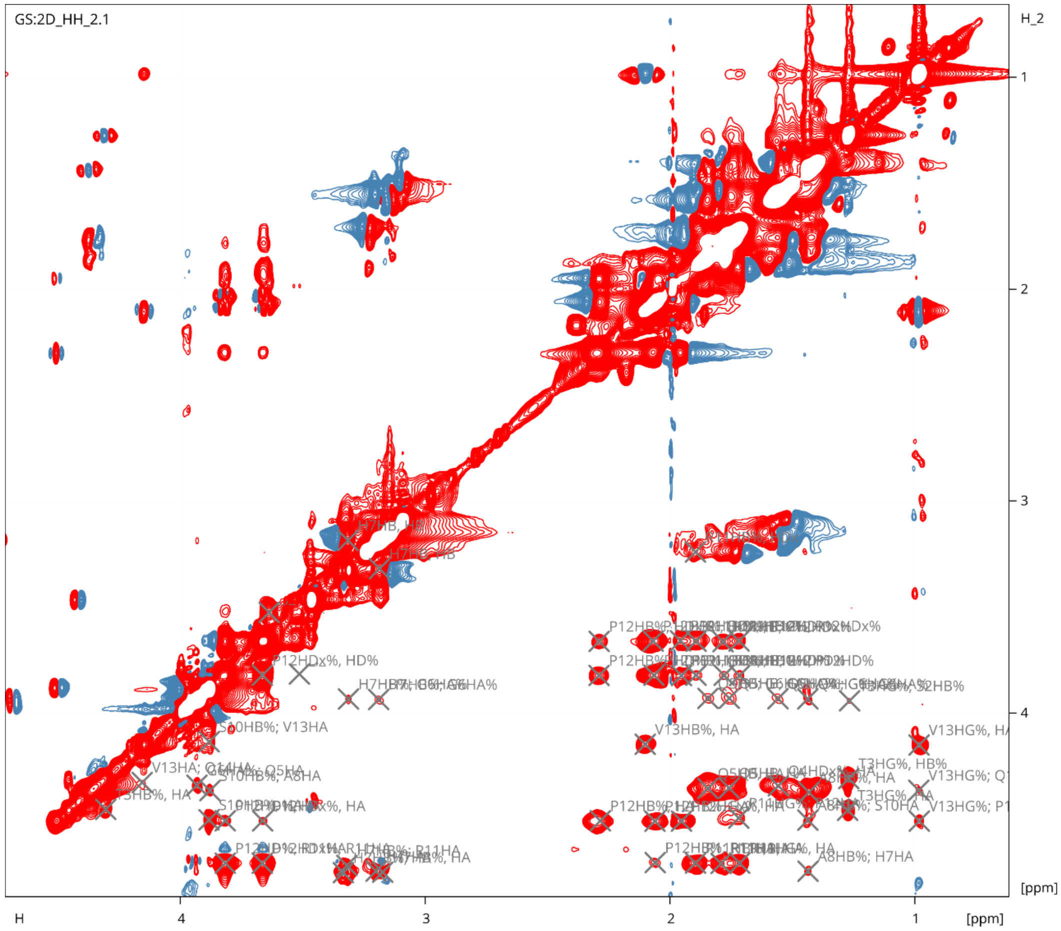

Figure S9. (Pages 23 and 24) Selected regions of the 2D NOESY spectrum of P10 peptide in a 70:30 (v/v) solution of H_2O/TFE-d2 at pH 3 (500 MHz, 298 K). The regions are displayed at different levels to make the lower-intensity correlation peaks more visible.

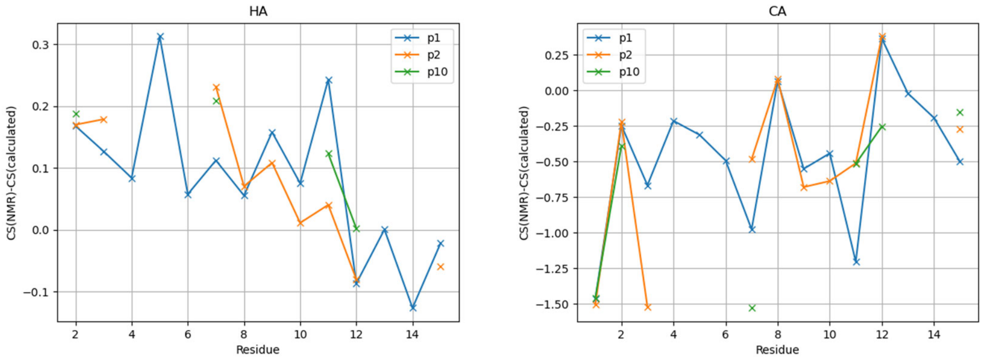

Figure S10. Chemical Shift (CS) difference between the experimental (NMR) and calculated CS from the peptide trajectories for the HA and the CA atoms. Sparta+ can only calculate the CS of residues without post-translational modifications, which is why some CS are missing.

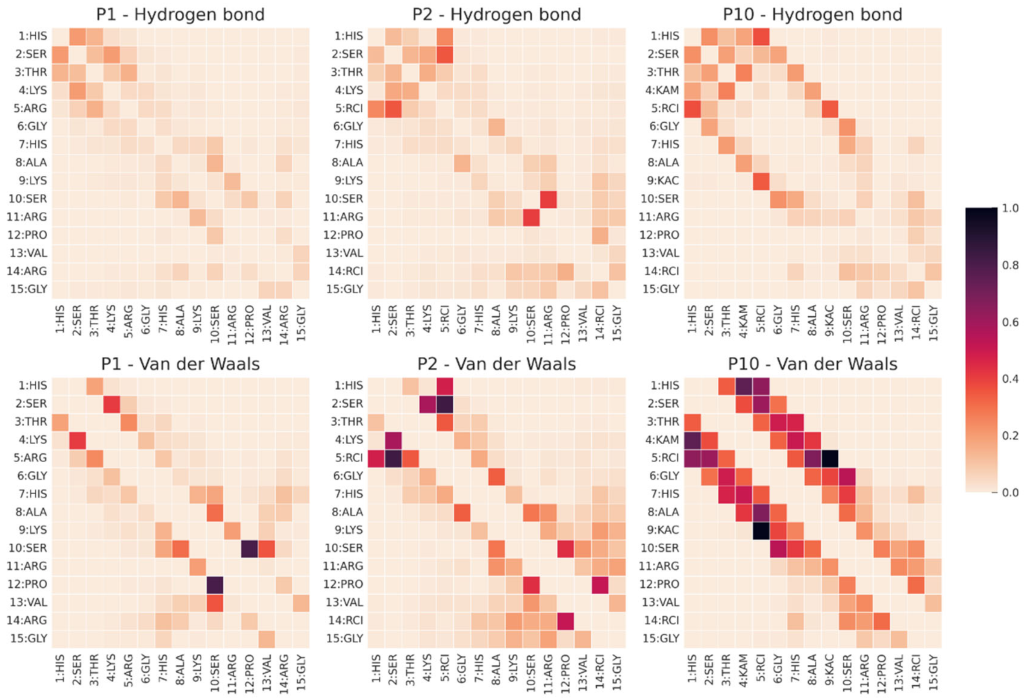

Figure S11. Intramolecular contacts arising from the Molecular Dynamics trajectories. The i,i+3 and i,i+4 contacts are typical of α-helices and more prevalent in P10. P2 shows more contacts than P1 because it is more compact, especially in the C-term region. This region is similar to P10 region, as expected because they share the same sequence.

Table S1. Peptides net charge and estimation of the α-helix content according to molar ellipticity at 222nm.

Peptide	Net charge (pH=7)	% α-helix
P1	+5	9
P2	+3	19
P3	+3	18
P4	+3	22
P5	+1	24
P6	+1	21
P7	+3	14
P8	+3	20
P9	+1	16
P10	+1	23
P11	+2	18

Table S2. P1 peptide NOE restraints in 30% TFE.

Assign F1	Assign F2	Pos F1	Pos F2	interval	classification
				Å	NOEs NMR
A.7.HIS.HB1	A.7.HIS.HB2	3.173	3.308		
A.7.HIS.HB2	A.7.HIS.HB1	3.308	3.173		
A.2.SER.H	A.1.HIS.HA	8.844	4.412	1.8-3.3	medium
A.2.SER.H	A.1.HIS.HB	8.844	3.456	> 5.5	very weak
A.3.THR.H	A.2.SER.HA	8.440	4.681	1.8-3.3	medium
A.2.SER.H	A.2.SER.HA	8.842	4.684	1.8-3.3	medium
A.3.THR.H	A.2.SER.HB	8.440	3.948	1.8-5.5	weak
A.2.SER.H	A.2.SER.HB	8.844	3.938	1.8-5.5	weak
A.2.SER.H	A.3.THR.H	8.845	8.441	1.8-5.5	weak
A.4.LYS.H	A.3.THR.HA	8.307	4.498	1.8-3.3	medium
A.3.THR.HG	A.3.THR.HA	1.260	4.498	1.8-5.5	weak
A.3.THR.HA	A.3.THR.HB	4.494	4.316	1.8-5.5	weak
A.3.THR.H	A.3.THR.HB	8.440	4.318	1.8-5.5	weak
A.3.THR.HG	A.3.THR.HB	1.259	4.315	1.8-3.3	medium
A.4.LYS.HB2	A.3.THR.HG	1.883	1.271	> 5.5	very weak
A.4.LYS.HD	A.3.THR.HG	1.710	1.259	> 5.5	very weak
A.3.THR.H	A.3.THR.HG	8.439	1.261	1.8-5.5	weak
A.4.LYS.H	A.3.THR.HG	8.304	1.261	1.8-5.5	weak
A.4.LYS.HG	A.3.THR.HG	1.501	1.276	1.8-5.5	weak
A.3.THR.H	A.4.LYS.H	8.438	8.307	1.8-3.3	medium
A.4.LYS.H	A.4.LYS.HA	8.291	4.376	1.8-2.5	strong
A.3.THR.HG	A.4.LYS.HA	1.250	4.382	> 5.5	very weak
A.2.SER.HB	A.4.LYS.HA	3.951	4.382	1.8-5.5	weak
A.4.LYS.H	A.4.LYS.HB2	8.293	1.890	1.8-5.5	weak
A.4.LYS.H	A.4.LYS.HB1	8.297	1.789	1.8-3.3	medium
A.4.LYS.H	A.4.LYS.HD	8.289	1.691	1.8-5.5	weak
A.4.LYS.H	A.4.LYS.HG	8.300	1.509	1.8-5.5	weak
A.5.ARG.HG	A.5.ARG.HA	1.719	4.692	1.8-5.5	weak
A.5.ARG.HB1	A.5.ARG.HA	1.777	4.690	1.8-3.3	medium
A.5.ARG.HB2	A.5.ARG.HA	1.878	4.685	1.8-5.5	weak
A.5.ARG.H	A.5.ARG.HB2	8.209	1.887	1.8-3.3	medium
A.5.ARG.H	A.5.ARG.HB1	8.209	1.780	1.8-3.3	medium
A.5.ARG.H	A.5.ARG.HD	8.208	3.229	1.8-5.5	weak
A.5.ARG.H	A.5.ARG.HG	8.208	1.743	1.8-5.5	weak
A.6.GLY.H	A.6.GLY.HA	8.288	3.955	1.8-3.3	medium
A.4.LYS.HD	A.6.GLY.HA	1.694	3.944	> 5.5	very weak
A.7.HIS.NHE	A.6.GLY.HA	7.318	3.951	1.8-5.5	weak
A.8.ALA.HB	A.6.GLY.HA	1.429	3.954	> 5.5	very weak
A.8.ALA.H	A.7.HIS.HA	8.330	4.749	1.8-3.3	medium
A.7.HIS.H	A.7.HIS.HA	8.290	4.746	1.8-3.3	medium
A.7.HIS.H	A.7.HIS.HB1	8.293	3.179	1.8-5.5	weak
A.7.HIS.HE	A.7.HIS.HB2	7.321	3.305	1.8-5.5	weak

A.7.HIS.HE	A.7.HIS.HB1	7.322	3.176	1.8-5.5	weak
A.7.HIS.H	A.8.ALA.HB	8.296	1.441	1.8-5.5	weak
A.8.ALA.H	A.7.HIS.HB2	8.330	3.308	1.8-5.5	weak
A.8.ALA.H	A.7.HIS.HB1	8.330	3.185	1.8-5.5	weak
A.7.HIS.H	A.7.HIS.HB2	8.293	3.304	1.8-5.5	weak
A.8.ALA.HB	A.8.ALA.HA	1.433	4.371	1.8-3.3	medium
A.7.HIS.HE	A.8.ALA.HA	7.322	4.378	1.8-5.5	weak
A.9.LYS.HB2	A.9.LYS.HA	1.889	4.391	1.8-3.3	medium
A.8.ALA.H	A.9.LYS.HG	8.330	1.439	1.8-5.5	weak
A.9.LYS.HB2	A.9.LYS.HA	1.784	4.380	1.8-3.3	medium
A.13.VAL.HA	A.9.LYS.HA	4.167	4.365	1.8-5.5	weak
A.10.SER.HB	A.9.LYS.HA	3.872	4.395	1.8-5.5	weak
A.10.SER.H	A.9.LYS.HA	8.117	4.399	1.8-2.5	strong
A.9.LYS.H	A.9.LYS.HB1	8.235	1.800	1.8-5.5	weak
A.9.LYS.H	A.9.LYS.HB2	8.232	1.906	1.8-5.5	weak
A.10.SER.H	A.9.LYS.HB2	8.117	1.905	1.8-5.5	weak
A.10.SER.H	A.9.LYS.HB1	8.118	1.796	1.8-5.5	weak
A.10.SER.H	A.9.LYS.HG2	8.117	1.515	1.8-5.5	weak
A.10.SER.H	A.9.LYS.HG1	8.117	1.440	1.8-5.5	weak
A.11.ARG.HB2	A.10.SER.HB	1.884	3.844	1.8-5.5	weak
A.11.ARG.HB1	A.10.SER.HB	1.789	3.861	1.8-5.5	weak
A.11.ARG.HG	A.10.SER.HB	1.721	3.858	> 5.5	very weak
A.11.ARG.H	A.9.LYS.HG1	8.209	1.437	> 5.5	very weak
A.11.ARG.H	A.9.LYS.HG2	8.212	1.513	> 5.5	very weak
A.10.SER.H	A.10.SER.HA	8.118	4.511	1.8-3.3	medium
A.13.VAL.HB	A.10.SER.HA	2.053	4.513	1.8-3.3	medium
A.11.ARG.H	A.10.SER.HA	8.208	4.511	1.8-2.5	strong
A.9.LYS.HG1	A.10.SER.HA	1.439	4.509	1.8-5.5	weak
A.9.LYS.HG1	A.10.SER.HB	1.447	3.880	> 5.5	very weak
A.10.SER.H	A.10.SER.HB	8.117	3.873	1.8-5.5	weak
A.11.ARG.H	A.10.SER.HB	8.208	3.873	1.8-5.5	weak
A.13.VAL.HB	A.11.ARG.HA	2.059	4.691	1.8-5.5	weak
A.12.PRO.HD2	A.11.ARG.HA	3.818	4.692	1.8-5.5	weak
A.12.PRO.HD1	A.11.ARG.HA	3.651	4.693	1.8-5.5	weak
A.11.ARG.HG	A.12.PRO.HA	1.714	4.508	1.8-5.5	weak
A.11.ARG.HB1	A.12.PRO.HA	1.785	4.506	1.8-5.5	weak
A.11.ARG.HB2	A.12.PRO.HA	1.885	4.511	1.8-5.5	weak
A.11.ARG.HB	A.11.ARG.HA	1.882	4.707	1.8-5.5	weak
A.10.SER.H	A.11.ARG.HA	8.119	4.691	1.8-5.5	weak
A.10.SER.HB	A.12.PRO.HA	3,874	4,509	1.8-5.5	weak
A.12.PRO.HB2	A.12.PRO.HA	2.278	4.512	1.8-3.3	medium
A.12.PRO.HD2	A.12.PRO.HA	3.819	4.511	1.8-5.5	weak
A.12.PRO.HD1	A.12.PRO.HA	3.646	4.513	1.8-5.5	weak
A.14.ARG.H	A.12.PRO.HB2	8.240	2.278	> 5.5	very weak
A.13.VAL.H	A.12.PRO.HB1	7.992	1.954	1.8-5.5	weak
A.13.VAL.H	A.12.PRO.HB2	7.992	2.280	1.8-5.5	weak

A.13.VAL.H	A.12.PRO.HD2	7.991	3.824	> 5.5	very weak
A.13.VAL.H	A.12.PRO.HD1	7.992	3.649	> 5.5	very weak
A.12.PRO.HB1	A.12.PRO.HD2	2.267	3.824	1.8-5.5	weak
A.12.PRO.HB1	A.12.PRO.HD2	1.968	3.823	1.8-5.5	weak
A.11.ARG.HG	A.12.PRO.HD1	1.723	3.647	> 5.5	very weak
A.11.ARG.HB1	A.12.PRO.HD2	1.791	3.822	> 5.5	very weak
A.11.ARG.HB2	A.12.PRO.HD2	1.880	3.823	> 5.5	very weak
A.11.ARG.HB2	A.12.PRO.HD2	1.881	3.646	> 5.5	very weak
A.11.ARG.HB1	A.12.PRO.HD2	1.793	3.647	1.8-5.5	weak
A.11.ARG.HG	A.12.PRO.HD1	1.723	3.647	> 5.5	very weak
A.12.PRO.HG	A.12.PRO.HD2	2.054	3.823	1.8-3.3	medium
A.12.PRO.HG	A.12.PRO.HD1	2.062	3.647	1.8-3.3	medium
A.12.PRO.HB1	A.12.PRO.HD1	1.969	3.645	1.8-3.3	medium
A.12.PRO.HB2	A.12.PRO.HD1	2.267	3.648	1.8-5.5	weak
A.11.ARG.H	A.12.PRO.HD1	8.208	3.649	> 5.5	very weak
A.11.ARG.H	A.12.PRO.HD2	8.208	3,820	1.8-5.5	weak
A.10.SER.H	A.13.VAL.H	8.118	8.009	1.8-5.5	weak
A.14.ARG.H	A.13.VAL.H	8.238	7.993	1.8-5.5	weak
A.13.VAL.HG	A.13.VAL.HA	0.974	4.166	1.8-5.5	weak
A.13.VAL.HB	A.13.VAL.HA	2.098	4.166	1.8-5.5	weak
A.13.VAL.H	A.13.VAL.HA	7.999	4.168	1.8-3.3	medium
A.14.ARG.HB1	A.13.VAL.HA	1.790	4.176	> 5.5	very weak
A.14.ARG.HB2	A.13.VAL.HA	1.897	4.175	1.8-5.5	weak
A.14.ARG.H	A.13.VAL.HA	8.237	4.166	1.8-3.3	medium
A.14.ARG.HG	A.13.VAL.HA	1.667	4.151	1.8-5.5	weak
A.13.VAL.H	A.13.VAL.HB	7.992	2.098	1.8-3.3	medium
A.14.ARG.H	A.13.VAL.HB	8.238	2.098	1.8-5.5	weak
A.13.VAL.H	A.13.VAL.HG	7.992	0.980	1.8-5.5	weak
A.14.ARG.H	A.13.VAL.HG	8.238	0.978	1.8-5.5	weak
A.13.VAL.H	A.14.ARG.HB1	7.996	1.786	> 5.5	very weak
A.13.VAL.H	A.14.ARG.HG	7.994	1.716	> 5.5	very weak
A.15.GLY.H	A.15.GLY.HA	8.336	3.957	1.8-3.3	medium
A.13.VAL.HG	A.14.ARG.HA	0.980	4.366	> 5.5	very weak

Table S3. P2 peptide NOE restraints in 30% TFE

Assign F1	Assign F2	Pos F1	Pos F2	interval	classification
				Å	NOEs NMR
P2.2.SER.H	P2.1.HIS.HA	8.870	4.421	1.8 - 5.5	weak
P2.1.HIS.HB2	P2.1.HIS.HB1	3.456	3.317	1.8-3.3	medium
P2.1.HIS.HB2	P2.1.HIS.HB1	3.458	3.350		
P2.2.SER.H	P2.1.HIS.HB2	8.870	3.465	> 5.5	very weak
P2.2.SER.H	P2.1.HIS.HD	8.872	8.693	1.8-5.5	weak
P2.2.SER.H	P2.1.HIS.HD	8.859	8.691		
P2.3.THR.H	P2.2.SER.HA	8.477	4.693	1.8-2.5	strong
P2.2.SER.H	P2.2.SER.HA	8.868	4.700	1.8-3.3	medium
P2.2.SER.H	P2.2.SER.HB1	8.871	3.921	1.8-5.5	weak
P2.2.SER.H	P2.2.SER.HB2	8.870	3.957	1.8-5.5	weak
P2.3.THR.H	P2.2.SER.HB1	8.480	3.927	1.8-5.5	weak
P2.3.THR.H	P2.2.SER.HB2	8.478	3.970	1.8-5.5	weak
P2.5.CIT.H	P2.3.THR.H	8.220	8.477	> 5.5	very weak
P2.2.SER.H	P2.3.THR.H	8.873	8.480	1.8-5.5	weak
P2.4.LYS.H	P2.3.THR.H	8.316	8.479	1.8-5.5	weak
P2.3.THR.HB	P2.3.THR.HA	4.321	4.484	1.8-5.5	weak
P2.3.THR.HG	P2.3.THR.HA	1.272	4.489	1.8-5.5	weak
P2.4.LYS.H	P2.3.THR.HA	8.316	4.488	1.8-3.3	medium
P2.3.THR.HG	P2.3.THR.HB	1.271	4.327	1.8-3.3	medium
P2.3.THR.H	P2.3.THR.HB	8.478	4.328	1.8-5.5	weak
P2.4.LYS.HG1	P2.3.THR.HG	1.444	1.274	1.8-5.5	weak
P2.4.LYS.HG1	P2.3.THR.HG	1.443	1.296		
P2.3.THR.HA	P2.3.THR.HG	4.488	1.276	1.8-5.5	weak
P2.5.CIT.H	P2.3.THR.HG	8.217	1.275	> 5.5	very weak
P2.4.LYS.H	P2.3.THR.HG	8.315	1.275	1.8-5.5	weak
P2.3.THR.H	P2.3.THR.HG	8.478	1.275	1.8-5.5	weak
P2.3.THR.H	P2.4.LYS.H	8.477	8.317	1.8-3.3	medium
P2.4.LYS.H	P2.4.LYS.HB1	8.315	1.875	1.8-5.5	weak
P2.4.LYS.H	P2.4.LYS.HB2	8.319	1.776	1.8-3.3	medium
P2.5.CIT.H	P2.4.LYS.HG1	8.223	1.455	1.8-5.5	weak
P2.4.LYS.H	P2.4.LYS.HG2	8.314	1.551	1.8-5.5	weak
P2.4.LYS.H	P2.5.CIT.H	8.301	8.233	1.8-2.5	strong
P2.3.THR.H	P2.5.CIT.H	8.472	8.236	1.8-3.3	medium
P2.5.CIT.HB1	P2.5.CIT.HA	1.768	4.372	1.8-3.3	medium
P2.5.CIT.H	P2.5.CIT.HA	8.216	4.375	1.8-2.5	strong
P2.2.SER.HB2	P2.5.CIT.HA	3.964	4.361	> 5.5	very weak
P2.2.SER.HB1	P2.5.CIT.HA	3.923	4.363	1.8-5.5	weak
P2.5.CIT.HG	P2.5.CIT.HB1	1.561	1.751	1.8-3.3	medium
P2.5.CIT.HG	P2.5.CIT.HB2	1.588	1.858	1.8-3.3	medium
P2.5.CIT.H	P2.5.CIT.HD	8.218	3.148	1.8-5.5	weak
P2.5.CIT.H	P2.5.CIT.HG	8.217	1.568	1.8-5.5	weak
P2.8.ALA.H	P2.6.GLY.HA1	8.345	3.930	1.8-3.3	medium

P2.8.ALA.H	P2.6.GLY.HA2	8.345	3.966	1.8-2.5	strong
P2.7.HIS.HB1	P2.6.GLY.HA	3.189	3.950	1.8-5.5	weak
P2.7.HIS.HB2	P2.6.GLY.HA	3.319	3.941	1.8-5.5	weak
P2.7.HIS.HB2	P2.10.SER.HB	3.311	3.894	> 5.5	very weak
P2.7.HIS.HB1	P2.10.SER.HB	3.200	3.896	> 5.5	very weak
P2.7.HIS.HE	P2.6.GLY.HA	7.331	3.956	> 5.5	very weak
P2.5.CIT.H	P2.6.GLY.HA	8.222	3.954	1.8-5.5	weak
P2.7.HIS.HE	P2.7.HIS.H	7.331	8.291	1.8-5.5	weak
P2.9.LYS.ENH3+	P2.7.HIS.H	7.624	8.290	1.8-5.5	weak
P2.8.ALA.H	P2.7.HIS.HA	8.353	4.754	1.8-3.3	medium
P2.7.HIS.H	P2.7.HIS.HA	8.282	4.755	1.8-3.3	medium
P2.7.HIS.HD	P2.7.HIS.HB1	8.621	3.165	> 5.5	very weak
P2.7.HIS.HE	P2.7.HIS.HB1	7.331	3.182	1.8-5.5	weak
P2.7.HIS.HD	P2.7.HIS.HB2	8.624	3.314	1.8-5.5	weak
P2.7.HIS.HE	P2.7.HIS.HB2	7.332	3.307	1.8-5.5	weak
P2.7.HIS.H	P2.7.HIS.HB1	8.287	3.182	1.8-5.5	weak
P2.7.HIS.H	P2.7.HIS.HB2	8.287	3.318	1.8-5.5	weak
P2.8.ALA.H	P2.7.HIS.HB1	8.353	3.189	1.8-5.5	weak
P2.8.ALA.H	P2.7.HIS.HB2	8.353	3.323	1.8-5.5	weak
P2.7.HIS.HB1	P2.7.HIS.HB2	3.181	3.323		
P2.7.HIS.HB2	P2.7.HIS.HB1	3.314	3.181		
P2.8.ALA.HB	P2.7.HIS.HB2	1.441	3.295	> 5.5	very weak
P2.8.ALA.HB	P2.7.HIS.HB1	1.437	3.195	> 5.5	very weak
P2.7.HIS.HE	P2.8.ALA.HA	7.330	4.377	1.8-5.5	weak
P2.7.HIS.HB1	P2.8.ALA.HA	3,199	4,378	> 5.5	very weak
P2.7.HIS.HB2	P2.8.ALA.HA	3.308	4.380	> 5.5	very weak
P2.9.LYS.H	P2.8.ALA.HA	8.334	4.377	1.8-2.5	strong
P2.8.ALA.HB	P2.8.ALA.HA	1.441	4.374	1.8-3.3	medium
P2.10.SER.HB	P2.8.ALA.HA	3.884	4.387	1.8-5.5	weak
P2.8.ALA.H	P2.8.ALA.HB	8.351	1.448	1.8-3.3	medium
P2.7.HIS.H	P2.8.ALA.HB	8.298	1.450	1.8-5.5	weak
P2.10.SER.H	P2.9.LYS.H	8.144	8.334	1.8-3.3	medium
P2.10.SER.H	P2.9.LYS.HA	8.144	4.395	1.8-2.5	strong
P2.10.SER.H	P2.9.LYS.HB1	8.145	1.805	1.8-5.5	weak
P2.10.SER.H	P2.9.LYS.HB2	8.145	1.915	1.8-5.5	weak
P2.9.LYS.H	P2.9.LYS.HB1	8.330	1.801	1.8-3.3	medium
P2.9.LYS.H	P2.9.LYS.HB2	8.331	1.905	1.8-5.5	weak
P2.10.SER.H	P2.9.LYS.HG1	8.144	1.450	1.8-5.5	weak
P2.10.SER.H	P2.9.LYS.HG2	8.145	1.507	1.8-5.5	weak
P2.9.LYS.H	P2.9.LYS.HG2	8.331	1.524	1.8-5.5	weak
P2.13.VAL.H	P2.10.SER.H	8,131	8,005	1.8-5.5	weak
P2.9.LYS.H	P2.10.SER.H	8.336	8.146	1.8-3.3	medium
P2.10.SER.H	P2.11.ARG.H	8.147	8.220	1.8-3.3	medium
P2.10.SER.H	P2.10.SER.HA	8.146	4.508	1.8-3.3	medium
P2.10.SER.HB	P2.10.SER.HA	3.886	4.506	1.8-3.3	medium
P2.8.ALA.HB	P2.10.SER.HA	1.443	4.503	1.8-5.5	weak

P2.11.ARG.H	P2.10.SER.HA	8.233	4.508	1.8-2.5	strong
P2.9.LYS.HD	P2.10.SER.HB	1.743	3.883	> 5.5	very weak
P2.10.SER.H	P2.10.SER.HB	8.144	3.885	1.8-5.5	weak
P2.11.ARG.H	P2.10.SER.HB	8.233	3.886	1.8-5.5	weak
P2.11.ARG.H	P2.11.ARG.HA	8.231	4.696	1.8-3.3	medium
P2.12.PRO.HD	P2.11.ARG.HA	3.674	4.697	1.8-5.5	weak
P2.12.PRO.HD	P2.11.ARG.HA	3.829	4.697	1.8-5.5	weak
P2.12.PRO.HG	P2.11.ARG.HA	2,068	4,695	1.8-5.5	weak
P2.11.ARG.HG	P2.11.ARG.HA	1.725	4.696	1.8-5.5	weak
P2.10.SER.H	P2.11.ARG.HA	8.145	4.698	1.8-5.5	weak
P2.14.CIT.HB2	P2.11.ARG.HA	1,898	4,677	1.8-5.5	weak
P2.11.ARG.HB2	P2.11.ARG.HA	1.899	4.717	1.8-5.5	weak
P2.11.ARG.H	P2.11.ARG.HD	8,234	3,245	> 5.5	very weak
P2.12.PRO.HD1	P2.12.PRO.HD2	3,673	3,836	1.8-2.5	strong
P2.12.PRO.HD1	P2.11.ARG.HD	3.669	3.248	> 5.5	very weak
P2.14.CIT.HB1	P2.12.PRO.HA	1.789	4.502	1.8-5.5	weak
P2.12.PRO.HG	P2.12.PRO.HA	2.061	4.505	1.8-5.5	weak
P2.12.PRO.HD2	P2.12.PRO.HA	3.825	4.505	1.8-5.5	weak
P2.12.PRO.HD1	P2.12.PRO.HA	3.672	4.505	1.8-5.5	weak
P2.13.VAL.H	P2.12.PRO.HA	8.015	4.505	1.8-2.5	strong
P2.14.CIT.H	P2.12.PRO.HB1	8.262	1.943	1.8-5.5	weak
P2.13.VAL.H	P2.12.PRO.HB1	8.014	1.954	1.8-5.5	weak
P2.13.VAL.H	P2.12.PRO.HB2	8.015	2.304	1.8-5.5	weak
P2.14.CIT.H	P2.12.PRO.HB2	8.262	2.299	> 5.5	very weak
P2.12.PRO.HB1	P2.12.PRO.HB2	1.969	2.296	1.8-2.5	strong
P2.12.PRO.HB1	P2.12.PRO.HD1	1.966	3.668	1.8-3.3	medium
P2.12.PRO.HB1	P2.12.PRO.HD1	1.944	3.669		
P2.12.PRO.HB1	P2.12.PRO.HD2	1.971	3.837	1.8-3.3	medium
P2.12.PRO.HB1	P2.12.PRO.HD2	1,952	3,837		
P2.14.CIT.HB1	P2.12.PRO.HD1	1.731	3.670	1.8-5.5	weak
P2.14.CIT.HB1	P2.12.PRO.HD1	1.792	3.670		
P2.14.CIT.HB1	P2.12.PRO.HD2	1.796	3.842	> 5.5	very weak
P2.14.CIT.HB1	P2.12.PRO.HD2	1.732	3.834		
P2.14.CIT.HB2	P2.12.PRO.HD2	1.894	3.838	> 5.5	very weak
P2.14.CIT.HB2	P2.12.PRO.HD1	1.896	3.668	1.8-5.5	weak
P2.11.ARG.H	P2.12.PRO.HD2	8.232	3.835	1.8-5.5	weak
P2.11.ARG.H	P2.12.PRO.HD1	8.233	3.672	1.8-5.5	weak
P2.13.VAL.H	P2.12.PRO.HD2	8.017	3.836	> 5.5	very weak
P2.13.VAL.H	P2.12.PRO.HD1	8.013	3.671	1.8-5.5	weak
P2.12.PRO.HB2	P2.12.PRO.HD2	2.288	3.837	1.8-3.3	medium
P2.12.PRO.HB2	P2.12.PRO.HD1	2.292	3.670	1.8-5.5	weak
P2.11.ARG.HD	P2.12.PRO.HD1	3.242	3.668	> 5.5	very weak
P2.12.PRO.HD1	P2.12.PRO.HD2	3.673	3.836	1.8-2.5	strong
P2.12.PRO.HG	P2.12.PRO.HD2	2.068	3.835	1.8-3.3	medium
P2.12.PRO.HG	P2.12.PRO.HD1	2.073	3.669	1.8-3.3	medium
P2.14.CIT.H	P2.13.VAL.H	8.265	8.017	1.8-3.3	medium

**P2.13.VAL.H**	**P2.13.VAL.HA**	**8.020**	**4.160**	**1.8-3.3**	**medium**
**P2.13.VAL.HB**	**P2.13.VAL.HA**	**2.106**	**4.158**	**1.8-3.3**	**medium**
**P2.14.CIT.H**	**P2.13.VAL.HA**	**8.263**	**4.159**	**1.8-2.5**	**strong**
**P2.13.VAL.HG**	**P2.13.VAL.HA**	**0.982**	**4.161**	**1.8-5.5**	**weak**
**P2.14.CIT.H**	**P2.13.VAL.HB**	**8.264**	**2.110**	**1.8-5.5**	**weak**
**P2.13.VAL.H**	**P2.13.VAL.HB**	**8.014**	**2.106**	**1.8-3.3**	**medium**
**P2.13.VAL.HA**	**P2.13.VAL.HG**	**4.158**	**0.984**	**1.8-5.5**	**weak**
**P2.14.CIT.H**	**P2.13.VAL.HG**	**8.264**	**0.985**	**> 5.5**	**very weak**
**P2.13.VAL.H**	**P2.13.VAL.HG**	**8.015**	**0.988**	**1.8-5.5**	**weak**
**P2.13.VAL.H**	**P2.14.CIT.H**	**8.014**	**8.264**	**1.8-3.3**	**medium**
**P2.13.VAL.H**	**P2.14.GLN.HA**	**8.015**	**4.355**	**1.8-5.5**	**weak**
**P2.14.GLN.H**	**P2.14.GLN.HA**	**8.274**	**4.365**	**1.8-3.3**	**medium**
**P2.13.VAL.H**	**P2.14.CIT.HB1**	**8.014**	**1.743**	**> 5.5**	**very weak**
**P2.14.CIT.H**	**P2.14.CIT.HB1**	**8.262**	**1.771**	**1.8-5.5**	**weak**
**P2.14.CIT.H**	**P2.14.CIT.HB2**	**8.265**	**1.872**	**1.8-5.5**	**weak**
**P2.14.CIT.H**	**P2.14.CIT.HG**	**8.274**	**1.574**	**1.8-5.5**	**weak**

Table S4. P10 peptide NOE restraints in 30% TFE

Assign F1	Assign F2	Pos F1	Pos F2	interval	classification
				Å	NOEs NMR
P10.7.HIS.HB1	P10.7.HIS.HB2	3.186	3.320		
P10.7.HIS.HB2	P10.7.HIS.HB1	3.315	3.186		
P10.2.SER.H	P10.1.HIS.HA	8.884	4.421	1.8-3.3	medium
P10.2.SER.H	P10.1.HIS.HB	8.885	3.468	> 5.5	very weak
P10.3.THR.H	P10.2.SER.H	8.476	8.897	1.8-5.5	weak
P10.3.THR.H	P10.2.SER.HA	8.482	4.692	1.8-3.3	medium
P10.2.SER.H	P10.2.SER.HA	8.883	4.702	1.8-5.5	weak
P10.3.THR.HG	P10.2.SER.HB	1.266	3.942	> 5.5	very weak
P10.2.SER.H	P10.2.SER.HB	8.885	3.943	1.8-5.5	weak
P10.3.THR.H	P10.2.SER.HB	8.481	3.947	1.8-5.5	weak
P10.4.hCIT.H	P10.3.THR.H	8.274	8.486	1.8-3.3	medium
P10.5.CIT.H	P10.3.THR.H	8.212	8.483	1.8-5.5	weak
P10.5.CIT.H	P10.3.THR.HA	8.210	4.451	1.8-3.3	medium
P10.4.hCIT.H	P10.3.THR.HA	8.274	4.459	1.8-3.3	medium
P10.3.THR.HG	P10.3.THR.HA	1.274	4.458	1.8-5.5	weak
P10.3.THR.HB	P10.3.THR.HA	4.305	4.454	1.8-5.5	weak
P10.3.THR.H	P10.3.THR.HA	8.482	4.459	1.8-3.3	medium
P10.3.THR.H	P10.3.THR.HB	8.484	4.312	1.8-3.3	medium
P10.3.THR.HG	P10.3.THR.HB	1.270	4.309	1.8-3.3	medium
P10.6.GLY.H	P10.3.THR.HG	8.319	1.277	1.8-5.5	weak
P10.4.hCIT.H	P10.3.THR.HG	8.275	1.275	1.8-5.5	weak
P10.5.CIT.H	P10.3.THR.HG	8.209	1.275	1.8-5.5	weak
P10.3.THR.H	P10.3.THR.HG	8.483	1.276	1.8-5.5	weak
P10.4.hCIT.HD	P10.4.hCIT.HA	1.557	4.343	1.8-5.5	weak
P10.4.hCIT.H	P10.4.hCIT.HB2	8.271	1.837	1.8-5.5	weak
P10.4.hCIT.H	P10.4.hCIT.HB1	8.269	1.754	1.8-3.3	medium
P10.4.hCIT.H	P10.4.hCIT.HG	8.276	1.423	1.8-5.5	weak
P10.5.CIT.HB1	P10.5.CIT.HA	1.758	4.353	1.8-3.3	medium
P10.5.CIT.HB2	P10.5.CIT.HA	1.845	4.355	1.8-3.3	medium
P10.6.GLY.H	P10.5.CIT.HA	8.307	4.340	1.8-2.5	strong
P10.6.GLY.HA	P10.5.CIT.HA	3.932	4.340	1.8-5.5	weak
P10.5.CIT.H	P10.5.GLN.HB2	8.209	1.853	1.8-3.3	medium
P10.5.CIT.H	P10.5.GLN.HB1	8.210	1.762	1.8-3.3	medium
P10.5.CIT.H	P10.5.CIT.HG	8.208	1.562	1.8-5.5	weak
P10.5.CIT.HG	P10.6.GLY.HA	1.559	3.932	1.8-5.5	weak
P10.5.CIT.HB1	P10.6.GLY.HA	1.758	3.930	1.8-5.5	weak
P10.5.CIT.HB2	P10.6.GLY.HA	1.845	3.931	1.8-5.5	weak
P10.7.HIS.HB1	P10.6.GLY.HA	3.190	3.936	1.8-5.5	weak
P10.7.HIS.HB2	P10.6.GLY.HA	3.313	3.934	1.8-5.5	weak
P10.6.GLY.H	P10.6.GLY.HA	8.308	3.934	1.8-2.5	strong
P10.7.HIS.H	P10.6.GLY.HA	8.242	3.935	1.8-2.5	strong
P10.4.hCIT.HG	P10.6.GLY.HA	1.437	3.933	1.8-5.5	weak

P10.7.HIS.HB1	P10.7.HIS.HA	3.173	4.750	1.8-3.3	medium
P10.8.ALA.HB	P10.7.HIS.HA	1.436	4.745	1.8-5.5	weak
P10.7.HIS.H	P10.7.HIS.HA	8.239	4.748	1.8-3.3	medium
P10.8.ALA.H	P10.7.HIS.HA	8.327	4.746	1.8-3.3	medium
P10.7.HIS.H	P10.7.HIS.HB2	8.240	3.324	1.8-5.5	weak
P10.7.HIS.H	P10.7.HIS.HB1	8.241	3.182	1.8-5.5	weak
P10.7.HIS.HD	P10.6.GLY.HA	8,616	3,935	> 5.5	very weak
P10.8.ALA.H	P10.7.HIS.HB2	8.326	3.327	1.8-5.5	weak
P10.8.ALA.H	P10.7.HIS.HB1	8.326	3.188	1.8-5.5	weak
P10.7.HIS.HE	P10.7.HIS.HB1	7.321	3.182	1.8-5.5	weak
P10.7.HIS.HE	P10.7.HIS.HB2	7.321	3.325	1.8-5.5	weak
P10.7.HIS.H	P10.7.HIS.HE	8.244	7.323	1.8-5.5	weak
P10.8.ALA.H	P10.8.ALA.HA	8.320	4.355	1.8-2.5	strong
P10.8.ALA.HB	P10.8.ALA.HA	1.433	4.373	1.8-3.3	medium
P10.10.SER.HB	P10.8.ALA.HA	3.879	4.365	1.8-5.5	weak
P10.8.ALA.H	P10.8.ALA.HB	8.327	1.439	1.8-3.3	medium
P10.8.ALA.HB	P10.10.SER.HA	1.437	4.505	1.8-5.5	weak
P10.10.SER.H	P10.8.ALA.HA	8.151	4.366	1.8-2.5	strong
P10.9.LYS.H	P10.9.LYS.HB2	8.306	1.849	1.8-5.5	weak
P10.9.LYS.H	P10.9.LYS.HB1	8.303	1.749	1.8-5.5	weak
P10.10.SER.H	P10.9.LYS.HG	8.149	1.551	1.8-5.5	weak
P10.9.LYS.H	P10.9.LYS.HG	8.310	1.569	1.8-5.5	weak
P10.10.SER.H	P10.9.LYS.HG	8.149	1.436	1.8-5.5	weak
P10.5.CIT.H	P10.9.LYS.HG	8.214	1.438	1.8-5.5	weak
P10.13.VAL.H	P10.14.CIT.H	8.029	8.261	1.8-3.3	medium
P10.10.SER.H	P10.10.SER.HA	8.145	4.506	1.8-2.5	strong
P10.10.SER.HB	P10.10.SER.HA	3.883	4.503	1.8-3.3	medium
P10.10.SER.H	P10.10.SER.HB	8.147	3.882	1.8-3.3	medium
P10.11.ARG.H	P10.11.ARG.HA	8.143	4.708	1.8-3.3	medium
P10.12.PRO.HD1	P10.11.ARG.HA	3.665	4.708	1.8-5.5	weak
P10.12.PRO.HD2	P10.11.ARG.HA	3.816	4.707	1.8-5.5	weak
P10.7.HIS.HB	P10.11.ARG.HA	3.320	4.718	1.8-5.5	weak
P10.11.ARG.HG	P10.11.ARG.HA	1.715	4.707	1.8-5.5	weak
P10.11.ARG.HB1	P10.11.ARG.HA	1.792	4.709	1.8-5.5	weak
P10.11.ARG.HB2	P10.11.ARG.HA	1.895	4.707	1.8-3.3	medium
P10.12.PRO.HB1	P10.11.ARG.HA	2.060	4.705	1.8-5.5	weak
P10.13.VAL.H	P10.11.ARG.HB1	8.029	1.774	1.8-5.5	weak
P10.13.VAL.H	P10.11.ARG.HG	8.029	1.729	1.8-5.5	weak
P10.11.ARG.H	P10.11.ARG.HB2	8.146	1.887	1.8-5.5	weak
P10.11.ARG.H	P10.11.ARG.HB1	8.145	1.775	1.8-3.3	medium
P10.11.ARG.H	P10.11.ARG.HD	8.144	3.227	1.8-5.5	weak
P10.11.ARG.HB2	P10.11.ARG.HD	1.896	3.241	1.8-5.5	weak
P10.11.ARG.H	P10.11.ARG.HG	8.144	1,725	1.8-5.5	weak
P10.13.VAL.H	P10.12.PRO.HA	8.029	4.510	1.8-2.5	strong
P10.12.PRO.HB2	P10.12.PRO.HA	2.285	4.508	1.8-2.5	strong
P10.12.PRO.HB1	P10.12.PRO.HA	2.061	4.510	1.8-3.3	medium

P10.12.PRO.HG	P10.12.PRO.HA	1.953	4.509	1.8-3.3	medium
P10.12.PRO.HD2	P10.12.PRO.HA	3.816	4.508	1.8-5.5	weak
P10.12.PRO.HD1	P10.12.PRO.HA	3.664	4.508	1.8-5.5	weak
P10.11.ARG.HG	P10.12.PRO.HA	1.719	4.495	1.8-5.5	weak
P10.13.VAL.HG	P10.12.PRO.HA	0.982	4.509	1.8-5.5	weak
P10.13.VAL.HG	P10.14.CIT.HA	0.984	4.364	1.8-5.5	weak
P10.13.VAL.H	P10.12.PRO.HB1	8.029	1.948	1.8-5.5	weak
P10.13.VAL.H	P10.12.PRO.HB2	8.030	2.301	1.8-5.5	weak
P10.14.CIT.H	P10.12.PRO.HB2	8.261	2.298	> 5.5	very weak
P10.14.CIT.H	P10.12.PRO.HB1	8.260	1.942	1.8-5.5	weak
P10.12.PRO.HB1	P10.12.PRO.HD2	2.065	3.822	1.8-3.3	medium
P10.12.PRO.HB1	P10.12.PRO.HD1	2.069	3.661	1.8-3.3	medium
P10.11.ARG.HB	P10.12.PRO.HD2	1.893	3.822	1.8-5.5	weak
P10.11.ARG.HB	P10.12.PRO.HD2	1.779	3.822	1.8-5.5	weak
P10.11.ARG.HB	P10.12.PRO.HD1	1.783	3.664	1.8-5.5	weak
P10.11.ARG.HB	P10.12.PRO.HD1	1.893	3.661	1.8-5.5	weak
P10.12.PRO.HB2	P10.12.PRO.HD2	2.290	3.823	1.8-5.5	weak
P10.12.PRO.HB2	P10.12.PRO.HD1	2.289	3.662	1.8-5.5	weak
P10.11.ARG.HG	P10.12.PRO.HD2	1.716	3.823	1.8-5.5	weak
P10.11.ARG.HG	P10.12.PRO.HD1	1.718	3.660	1.8-5.5	weak
P10.12.PRO.HD1	P10.12.PRO.HD2	3.665	3.822	1.8-2.5	strong
P10.13.VAL.H	P10.12.PRO.HD2	8.032	3.822	> 5.5	very weak
P10.13.VAL.H	P10.12.PRO.HD1	8.028	3.661	1.8-5.5	weak
P10.11.ARG.H	P10.12.PRO.HD2	8.144	3.821	1.8-5.5	weak
P10.11.ARG.H	P10.12.PRO.HD1	8.145	3.661	1.8-5.5	weak
P10.12.PRO.HG	P10.12.PRO.HD1	1.957	3.659	1.8-3.3	medium
P10.12.PRO.HG	P10.12.PRO.HD2	1.953	3.823	1.8-3.3	medium
P10.9.LYS.ENH	P10.13.VAL.H	7.747	8.034	1.8-5.5	weak
P10.14.GLN.H	P10.13.VAL.H	8.259	8.033	1.8-3.3	medium
P10.10.SER.HB	P10.13.VAL.HA	3.882	4.135	1.8-5.5	weak
P10.13.VAL.HB	P10.13.VAL.HA	2.101	4.147	1.8-3.3	medium
P10.13.VAL.H	P10.13.VAL.HA	8.032	4.152	1.8-3.3	medium
P10.14.CIT.H	P10.13.VAL.HA	8.260	4.150	1.8-2.5	strong
P10.13.VAL.HG	P10.13.VAL.HA	0.983	4.150	1.8-5.5	weak
P10.13.VAL.H	P10.13.VAL.HB	8.029	2.102	1.8-3.3	medium
P10.14.CIT.H	P10.13.VAL.HB	8.259	2.104	1.8-5.5	weak
P10.14.CIT.H	P10.13.VAL.HG	8.260	0.984	1.8-5.5	weak
P10.13.VAL.H	P10.13.VAL.HG	8.030	0.988	1.8-5.5	weak
P10.13.VAL.HA	P10.14.CIT.HA	4.155	4.327	1.8-5.5	weak
P10.13.VAL.H	P10.14.CIT.HA	8.033	4.363	1.8-5.5	weak
P10.13.VAL.HG	P10.14.CIT.HA	0.984	4.364	> 5.5	very weak
